# Lipid Droplet‐Driven Ribosome Collisions Trigger ZAKα‐p38 Signaling to Accelerate Testicular Aging

**DOI:** 10.1111/acel.70359

**Published:** 2026-01-02

**Authors:** Yanghua Xu, Xiaoyan Shi, Yinghao Yin, Xiaoli Tan, Ningjing Ou, Xiaopeng Tang, Biao Liu, Hongshan Xie, Yuzhuo Chen, Zhitao Han, Jiarong Xu, Zitaiyu Li, Xiaoping Zheng, Hongji Hu, Wenjing Wang, Wanyi Xia, Hao Chen, Yuxin Tang, Liangyu Zhao

**Affiliations:** ^1^ Department of Urology The Fifth Affiliated Hospital, Sun Yat‐Sen University Zhuhai Guangdong China; ^2^ Department of Interventional Medicine, Guangdong Provincial Key Laboratory of Biomedical Imaging The Fifth Affiliated Hospital, Sun Yat‐Sen University Zhuhai Guangdong China; ^3^ Department of Human Cell Biology and Genetics, Joint Laboratory of Guangdong & Hong Kong Universities for Vascular Homeostasis and Diseases, School of Medicine; Shenzhen Key Laboratory of Gene Regulation and Systems Biology Southern University of Science and Technology Shenzhen China; ^4^ Department of Biological Sciences The University of Edinburgh Edinburgh Scotland UK; ^5^ School of Biomedical and Pharmaceutical Sciences Guangdong University of Technology Guangzhou China; ^6^ Department of Ultrasound The Fifth Affiliated Hospital, Sun Yat‐Sen University Zhuhai Guangdong China; ^7^ Department of Biochemistry, SUSTech Homeostatic Medicine Institute, School of Medicine Southern University of Science and Technology Shenzhen China; ^8^ Department of Traditional Chinese Medicine The Fifth Affiliated Hospital, Sun Yat‐Sen University Zhuhai Guangdong China

**Keywords:** lipid droplet, ribosome collisions, ROS, Sertoli cells, testicular aging

## Abstract

Testicular aging, a key feature of late‐onset hypogonadism (LOH), is closely associated with Sertoli cells dysfunction. Emerging evidence implicates lipid droplet (LD) accumulation as a hallmark of aging in Sertoli cells, but its role in Sertoli cells senescence and the associated molecular mechanisms are unknown. We found that aging and obesity drove progressive LD accumulation in Sertoli cells, accompanied by mitochondrial dysfunction and ROS overproduction. Palmitic Acid (PA)‐induced LD overload in vitro replicated these aging phenotypes, triggering ROS overproduction that provoked ribosome collisions and caused decreased protein synthesis globally. Moreover, LD‐driven ROS disrupted mRNA translation, particularly at GA‐rich sequences encoding aspartate and glutamate. Collided ribosomes activated the ZAKα‐p38 axis in Sertoli cells, causing cellular senescence and impairing the blood‐testis barrier. ZAKα inhibitor Nilotinib attenuated testicular atrophy, restored testosterone levels, and mitigated Sertoli cells dysfunction in aged mice. Targeting this pathway with ZAKα inhibitor offers a therapeutic strategy for age‐related gonadal decline, bridging lipid metabolism dysfunction, and reproductive aging.

## Graphical abstract

Aging‐ and obesity‐driven lipid droplet accumulation in Sertoli cells induces ROS‐mediated ribosome collisions, activating the ZAKα‐p38 pathway to accelerate testicular aging. Pharmacological ZAKα inhibition with Nilotinib rescues age‐related testicular dysfunction, identifying ribotoxic stress as a therapeutic target.

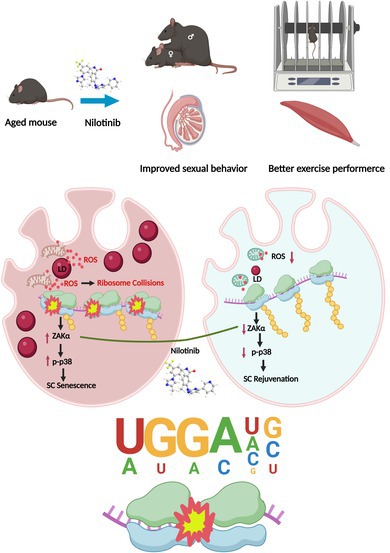

## 1 Introduction

The testes, vital organs responsible for spermatogenesis and testosterone secretion, play a central role in systemic health, with their functional decline directly impacting multiple physiological systems (Li, Lin, et al. 2024). Testicular aging is characterized by reduced testosterone production, structural degeneration, and the onset of late‐onset hypogonadism (LOH), a prevalent age‐related androgen deficiency disorder (Dong et al. 2022; Nieschlag 2020; Santiago et al. 2019; Zhao, Zhao, et al. 2023). LOH manifests clinically through hypogonadism‐associated symptoms such as osteoporosis, muscle atrophy, depressive states, erectile dysfunction, and impaired spermatogenesis (Jaschke et al. 2021; Rochira 2020; Snyder 2022). Despite its clinical significance, the molecular underpinnings of LOH remain poorly elucidated. Recent work revealed that Sertoli cells in LOH patients exhibit lysosomal acidification defects and pathological lipid droplet (LD) accumulation (Deng et al. 2024; Zhao, Li, et al. 2023). However, the mechanistic link between LD accumulation and Sertoli cell failure remains unresolved.

Lipid droplets, organelles comprising a neutral lipid core (triacylglycerols and cholesteryl esters) enveloped by a phospholipid monolayer, are increasingly recognized as mediators of cellular dysfunction when aberrantly accumulated in non‐adipose tissues (Olzmann and Carvalho 2019; Zadoorian et al. 2023). In neurodegenerative disorders, glial LD deposition exacerbates neuronal damage, while LD reduction ameliorates disease progression (Liu et al. 2015; Marschallinger et al. 2020; Mi et al. 2023). Similarly, renal medullary LD accumulation drives fibrosis in chronic kidney disease, and hepatic steatosis with LD overload is a hallmark of nonalcoholic fatty liver disease (Gluchowski et al. 2017; Kim et al. 2024; Mitrofanova et al. 2023). Mechanistically, excessive LDs impair mitochondrial function by overwhelming β‐oxidation capacity, leading to toxic free fatty acid spillover, mitochondrial dysfunction, and reactive oxygen species (ROS) overproduction (Liu et al. 2015).

Protein synthesis, a cornerstone of cellular homeostasis, becomes dysregulated during aging, with global translational attenuation contributing to organ dysfunction (Charmpilas et al. 2015; Jia et al. 2024; Stein et al. 2022). Ribosome collision, a hallmark of translational stress, arises from diverse insults including nutrient deprivation, mRNA structural anomalies, and oxidative damage (Misra et al. 2024). Collided ribosomes activate the ribotoxic stress response (RSR), wherein the sensor kinase ZAKα phosphorylates downstream effectors p38 and JNK, triggering either adaptive signaling or apoptotic cascades under sustained stress (Snieckute et al. 2022; Wu et al. 2020). Although ribosome collision‐dependent activation of ZAKα and downstream p38 has been characterized in liver and skin, its existence and functional significance in the reproductive system have not been described, highlighting the need to investigate this pathway in testicular aging (Sinha et al. 2024; Snieckute et al. 2023).

Here, we demonstrated that aging drove progressive LD accumulation in murine Sertoli cells, a process exacerbated by obesity and linked to accelerated testicular aging. In vitro, palmitic acid (PA)‐induced LD overload in primary Sertoli cells recapitulated key aging phenotypes, including mitochondrial dysfunction and ROS overproduction. Sustained ROS generation triggered ribosome collision, activating the ZAKα‐p38 axis and driving Sertoli cell dysfunction and senescence. Pharmacological inhibition of ZAKα with Nilotinib rescued age‐associated testicular decline in vivo, unveiling a previously unrecognized nexus between lipidotoxicity, translational stress, and gonadal aging. These findings position ribotoxic stress as a mechanistic bridge linking lipid metabolism dysfunction to Sertoli cell senescence, offering a therapeutic avenue for alleviating testicular aging.

## 2 Results

### 2.1 Obesity Exacerbates Testicular Aging In Vivo

**FIGURE 1:**
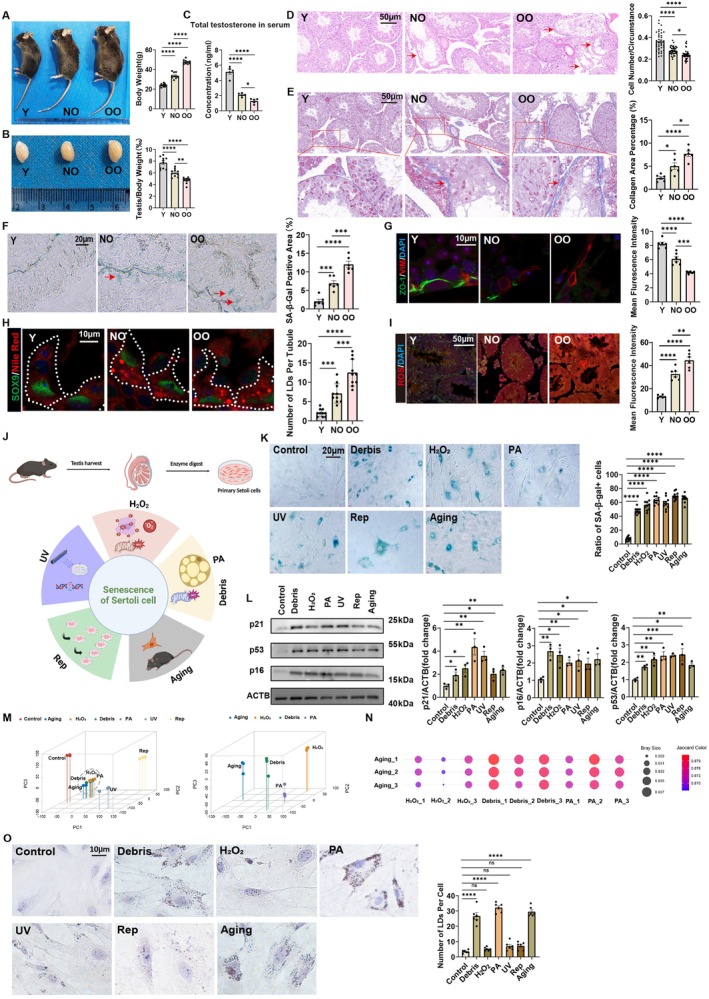
Obesity exacerbates testicular aging in vivo and in vitro (A) Representative images of mice in three experimental groups: Y (young, 2 months old), NO (naturally old, 20 months old), and OO (obese old, 20 months old). Body weight measurement of mice. *n* = 10. (B) Representative images of testis in three experimental groups. Testis‐to‐body weight ratio across experimental groups. *n* = 10. (C) Total serum testosterone concentration in three experimental groups. *n* = 5. (D) Representative images of HE staining of testicular tissue sections. Red arrows indicate disorganized tubules. *n* = 40. Scale bar = 50 μm. (E) Representative images of Masson staining of testicular tissue sections. Red arrows indicate accumulation of collagen fibers. *n* = 6. Scale bar = 50 μm. (F) Representative images of SA‐β‐gal staining of testicular tissue sections. Red arrows indicate SA‐β‐gal‐positive signals. *n* = 6. Scale bar = 20 μm. (G) Immunofluorescence staining for VIM (red) and ZO‐1 (green) in testicular tissue sections. *n* = 6. Scale bar = 10 μm. (H) Immunofluorescence staining for Nile Red (red) and SOX9 (green) in testicular tissue sections. *n* = 10. Scale bar = 10 μm. (I) Immunofluorescence staining for cellROX (red) and DAPI (blue) in testicular tissue sections. *n* = 6. Scale bar = 50 μm. (J) Schematic illustration of the experimental design. Primary Sertoli cells were isolated from testicular tissue. Multiple primary Sertoli cells aging models were employed, including PA (150 μM palmitic acid for 24 h), H_2_O_2_ (100 μM hydrogen peroxide for 2 h), UV (50 mJ/cm^2^ UV‐B irradiation), Debris (cell debris‐induced metabolic stress), Rep (replicative senescence induced by continuous passaging), and Aging (natural 20 months aging model). (K) SA‐β‐gal staining of Sertoli cells from different treatment groups. *n* = 10. Scale bar = 20 μm. (L) Western blot shows the level of p21, p53, p16 in Sertoli cells from different treatment groups. *n* = 3 technical repetitions. (M‐N) Principal component analysis (PCA) of gene expression from different treatment groups. Scatter plot visualization of Bray–Curtis dissimilarity indices across experimental samples (Debris, H_2_O_2_, PA, Aging). (O) Oil Red O staining of lipid accumulation in Sertoli cells from the different experimental groups. Scale bar = 10 μm. Bars represent means ± SEM. Statistical significance was assessed using one‐way ANOVA followed by Tukey's post hoc tests or Kruskal–Wallis test with Dunn post hoc tests. *p < 0.05, **p < 0.01, ***p < 0.001, ****p < 0.0001, ns, not significant.

To investigate testicular aging‐related alterations and determine whether obesity accelerates testicular aging, we established three experimental murine models: a young control group (2‐month‐old, Y), a naturally old group (20‐month‐old, NO), and an obese‐old group (20‐month‐old with obesity comorbidity, OO). The OO group exhibited a significant increase in body weight compared to Y and NO groups (Figure 1A), accompanied by a marked reduction in testis‐to‐body weight ratio (Figure 1B). Total serum testosterone levels were profoundly diminished in both NO and OO groups relative to Y controls, with the OO cohort displaying the most severe decline (Figure 1C). Histopathological analysis revealed pronounced testicular degeneration in OO mice, characterized by disorganized seminiferous tubules (HE staining) and excessive collagen deposition (Masson staining), phenotypes that were more severe than those observed in NO mice (Figure 1D,E). Besides, the Y group had the highest C/C score (cell number to lumen circumference ratio), significantly surpassing both the NO and OO groups, with the OO group having the lowest (Figure 1D). Immunofluorescence staining of the testes further revealed a significant number reduction in Leydig cells (LCs), Sertoli cells (SCs), and germ cells (GCs) in the NO and OO groups compared to the Y group (Figure S1A–C). Histopathological analysis of muscle, liver, and kidney in three experimental models (Y, NO, and OO) showed progressive tissue degeneration in the NO and OO groups, with OO showing more severe degeneration (Figure S1D,E). Notably, compared with Y testes, NO and OO testes exhibited elevated senescence‐associated β‐galactosidase activity, with OO testes showing the highest SA‐β‐gal activity in Sertoli cells, indicating Sertoli cells senescence (Figure 1F). As Sertoli cells are an integral component of the blood‐testis barrier, we examined the ZO‐1 protein, which is part of this barrier. Fluorescence staining of the ZO‐1 protein showed that, compared to Y group mice, the blood‐testis barrier integrity was compromised in NO and OO group mice, with the OO group being the most severely affected (Figure 1G). Besides, Nile Red and Oil Red O staining revealed accelerated lipid accumulation in NO and OO testes, with OO testes showing more pronounced lipid droplet accumulation (Figure 1H and Figure S1F). So, what is the connection between lipid droplet accumulation in testicular Sertoli cells and testicular aging? It has been reported that lipid droplet accumulation in microglia in the brain causes excessive ROS production, leading to brain aging (Marschallinger et al. 2020). Therefore, we also detected ROS in testicular tissue and found that ROS levels showed an age‐related increase. Compared to the Y and NO groups, the OO group exhibited significantly higher ROS levels (Figure 1I). The observed increase in ROS levels occurs broadly within the testicular tissue, affecting both Sertoli and germ cells. These findings position lipid‐driven ROS as a nexus connecting obesity and testicular aging, with comorbid obesity acting as a metabolic accelerant of age‐related gonadal decline in vivo.

### 2.2 Lipid Droplet Accumulation Triggers Sertoli Cells Senescence In Vitro

Due to the challenges of acquiring naturally aged Sertoli cells for mechanistic investigations, there is an urgent need to establish an in vitro model capable of recapitulating the hallmarks of naturally aged Sertoli cells. Firstly, we employed a two‐step enzymatic digestion procedure to isolate Sertoli cells and Leydig cells, achieving a final purity exceeding 90% (Figure S1G,H). Then, to systematically evaluate senescence phenotypes in Sertoli cells under varied aging paradigms, we established six distinct aging models: palmitic acid (PA)‐induced lipotoxicity, hydrogen peroxide (H_2_O_2_)‐mediated oxidative stress, ultraviolet (UV)‐triggered DNA damage, cell debris (Debris)‐driven metabolic stress, replicative senescence (Rep) via serial passaging, and natural aging (Aging, 20‐month‐old mice) (Figure 1J). SA‐β‐gal staining revealed a significant increase in SA‐β‐gal activity across all models compared to control (Figure 1K). Besides, immunoblotting analysis also demonstrated upregulated expression of senescence‐associated markers p53, p21, and p16 compared to control (Figure 1L). This indicates that all these modeling approaches can induce Sertoli cell senescence, while the underlying mechanisms may vary. Therefore, we performed RNA sequencing on these senescence‐induced Sertoli cells to identify which senescence patterns of RNA transcription most closely resemble natural aging. Principal component analysis (PCA) of transcriptomic profiles revealed close clustering of H_2_O_2_, Debris, and PA groups with the natural aging (Aging) cohort in 3D multidimensional space (PC1/PC2/PC3) (Figure 1M), suggesting shared molecular signatures between these aging models and physiological senescence. Moreover, Bray–Curtis dissimilarity and Jaccard indices demonstrated significantly higher overlap between Aging and the PA groups compared to H_2_O_2_ models, implicating conserved pathway activation across lipid accumulation‐induced aging and natural aging (Figure 1N). Lipid accumulation, assessed by Oil Red O staining, was markedly elevated in PA‐treated and naturally aged Sertoli cells, implicating lipid accumulation as a central driver of physiological senescence (Figure 1O). While H_2_O_2_ causes Sertoli cell senescence, it does not lead to lipid droplet accumulation. In contrast, the PA group induces cellular senescence and lipid droplet accumulation, similar to the aging group. These results demonstrate that lipid droplet‐driven senescence serves as a reliable in vitro model for natural aging, sharing conserved transcriptional networks and functional decline. Therefore, we will use the PA group in place of the natural aging group for subsequent studies.

### 2.3 Lipid Droplets in Sertoli Cells Damage Mitochondria, Causing ROS Production and Testicular Aging

**FIGURE 2:**
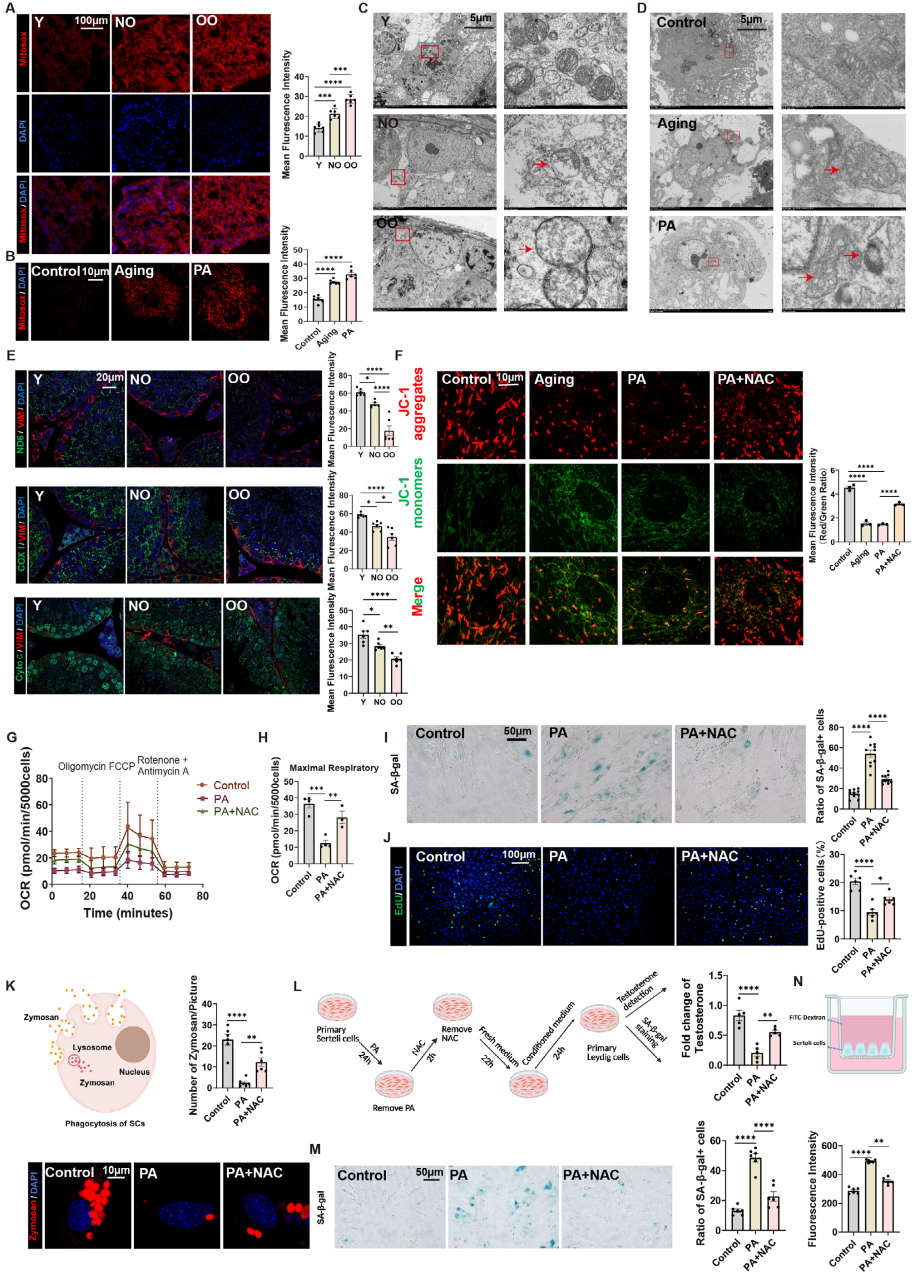
Mitochondrial damage‐induced ROS is a major cause of testicular aging (A, B) Immunofluorescence staining for Mitosox (red) and DAPI (blue) in testicular tissue sections and primary Sertoli cells. *n* = 6. Scale bar = 100 μm (upper panel), Scale bar = 10 μm (upper panel). (C, D) Representative images show mitochondria in Sertoli cells of testicular tissues and primary Sertoli cells using transmission electron microscopy (TEM). Red arrows indicate mitochondrial swelling. Scale bar = 10 μm. (E) Immunofluorescence staining for VIM (red), DAPI (blue) and ND6 (green), COX I (green), Cyto C (green) in testicular tissue sections. *n* = 6. Scale bar = 20 μm. (F) Fluorescence microscopy images of primary Sertoli cells stained with JC‐1 dye in four experimental conditions. *n* = 3. Scale bar = 10 μm. (G, H) Oxygen consumption rate (OCR) measurement of Sertoli cells from Control, PA, and NAC groups. Maximal respiratory capacity of Sertoli cells from NC, PA, and NAC groups, as determined by OCR. *n* = 3–4. (I) SA‐β‐gal staining of primary Sertoli cells from different experiments. *n* = 10. Scale bar = 50 μm. (J) EdU incorporation assay in Sertoli cells from NC, PA, and NAC groups to measure cell proliferation. *n* = 6. Scale bar = 100 μm. (K) Schematic illustration of Zymosan particle phagocytosis by Sertoli cells. Representative fluorescence images showing Zymosan particles (red) and nuclei (blue) in different group. *n* = 6. Scale bar = 10 μm. (L) Schematic workflow for evaluating testosterone secretion and SA‐β‐gal in Leydig cells exposed to conditioned media from treated Sertoli cells (Control, PA, NAC). Testosterone were quantified via ELISA. *n* = 5. (M) SA‐β‐gal staining of Leydig cells exposed to conditioned media from treated Sertoli cells (Control, PA, NAC). *n* = 6. Scale bar = 50 μm. (N) Schematic representation of Sertoli cells cultured with FITC‐Dextran. Bar graph quantifying FITC‐Dextran fluorescence intensity across experimental groups (Control, PA, NAC). *n* = 5. Bars represent means ± SEM. Statistical significance was assessed using one‐way ANOVA followed by Tukey's post hoc tests or Kruskal–Wallis test with Dunn post hoc tests. *p < 0.05, **p < 0.01, ***p < 0.001, ****p < 0.0001.

In vivo studies demonstrated that senescent Sertoli cells exhibited significantly elevated levels of reactive oxygen species (ROS), identifying ROS as a primary causative factor in aging (Figure 1I). Given that mitochondria represent the predominant intracellular source of ROS, we quantified mitochondrial ROS (Mitosox) levels, which revealed marked increases in Mitosox fluorescence in aged testicular tissues, senescent Sertoli cells, and PA‐induced senescence model (Figure 2A,B). Ultrastructural examination via transmission electron microscopy (TEM) demonstrated mitochondrial swelling in Sertoli cells of the testicular tissue in the NO and OO groups compared to the Y group, indicative of mitochondrial dysfunction (Figure 2C). Moreover, TEM also uncovered ultrastructural anomalies, including mitochondrial swelling and cristae disorganization in aging and PA‐treated Sertoli cells in vitro (Figure 2D). Concurrently, aged testes exhibited progressive declines in mitochondrial‐encoded proteins ND6 and cytochrome c oxidase subunit I (COX I), alongside reduced cytochrome c (Cyto C) levels, with maximal suppression in the OO group (Figure 2E). JC‐1 staining revealed mitochondrial membrane depolarization in aging and PA‐treated groups compared to controls, while N‐acetylcysteine (NAC)‐an ROS scavenger co‐treatment partially restored mitochondrial membrane depolarization (Figure 2F). PA treatment significantly reduced OCR and maximal respiratory capacity, indicating mitochondrial dysfunction. NAC co‐treatment partially restored OCR, suggesting its protective effect against PA‐induced mitochondrial damage (Figure 2G,H). PA treatment also markedly elevated SA‐β‐gal positive cells, whereas NAC co‐treatment attenuated PA‐triggered cellular senescence (Figure 2I). Moreover, PA significantly reduced cell proliferation by decreasing the percentage of EdU positive cells, while NAC co‐treatment reversed this inhibition and restored proliferative capacity (Figure 2J). PA treatment also significantly impaired phagocytic activity of Sertoli cells, demonstrated by decreased zymosan particle uptake compared to controls, while NAC co‐treatment partially reversed PA‐induced phagocytosis suppression (Figure 2K). We also investigated the effects of Sertoli cells senescence on Leydig cells function by co‐culturing Leydig cells with conditioned media from Sertoli cells subjected to different treatments. The results showed that conditioned media from senescent Sertoli cells impaired testosterone secretion in Leydig cells and promoted their senescence. However, treatment with NAC reduced these adverse effects (Figure 2L,M). PA also disrupted the blood‐testis barrier integrity by increasing permeability, as indicated by elevated FITC‐dextran fluorescence intensity. NAC treatment significantly reduced this PA‐induced increase in permeability, suggesting a protective role in restoring barrier function (Figure 2N). Together, these findings establish mitochondrial damage‐induced ROS as a central orchestrator of testicular aging, driving Sertoli cells senescence and its dysfunction while ROS‐scavenging NAC can partially reverse it.

### 2.4 ROS Causes a Global Decrease in Protein Synthesis due to Ribosome Collisions

**FIGURE 3:**
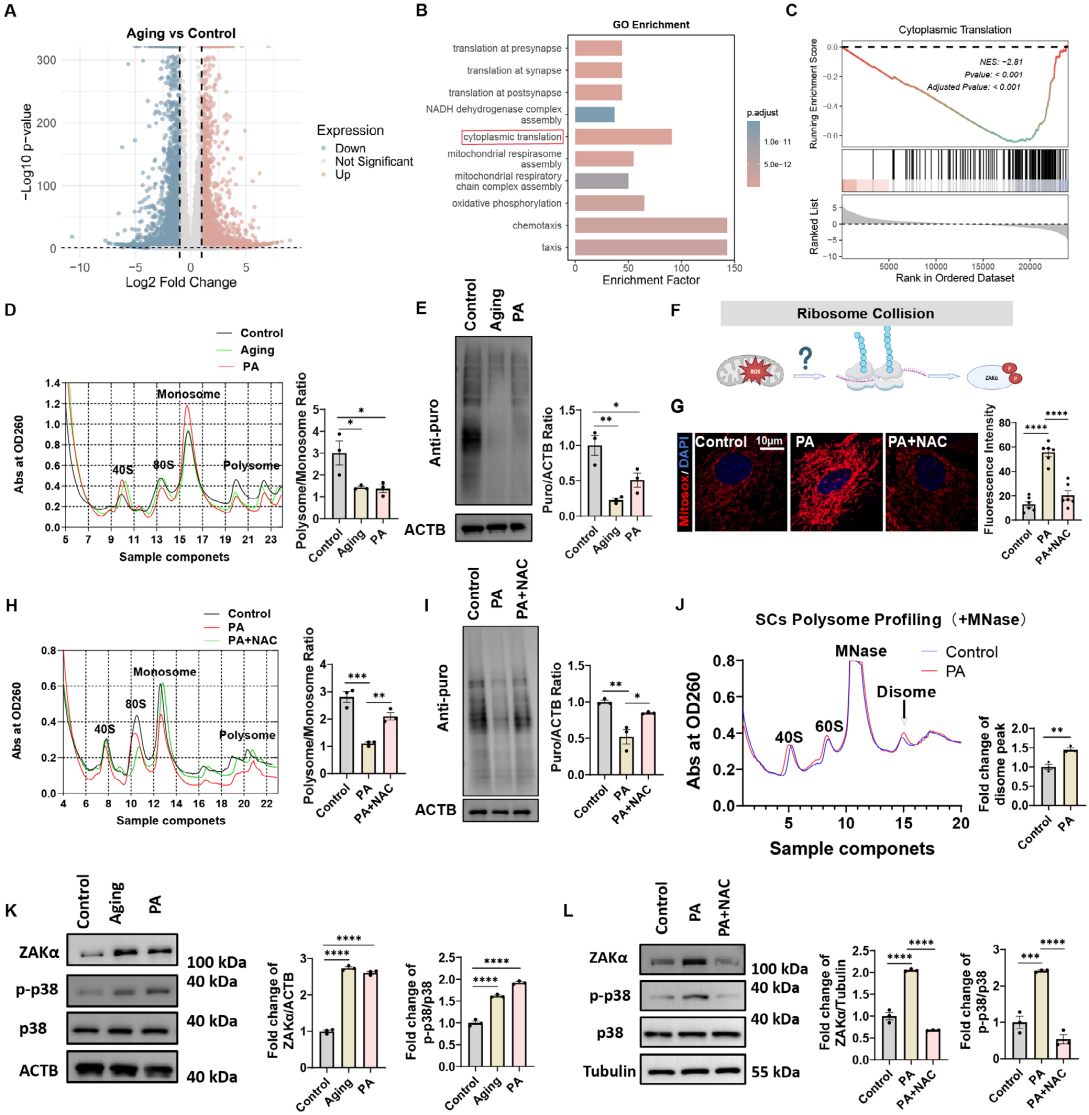
ROS causes a global decrease in protein synthesis due to ribosome collisions (A) Volcano plot of RNA‐seq data comparing Aging and Control groups. The downregulated genes are colored in blue, while the upregulated genes are in pink. (B) Gene ontology (GO) enrichment analysis of differentially regulated genes from RNA‐seq data comparing Aging and Control groups. (C) GSEA analysis of the “Cytoplasmic Translation” pathway. (D) Polysome profiling showed the change of global translation of different experimental groups in Sertoli cells. *n* = 3 technical repetitions. (E) Western blot analysis for puromycin incorporation in Sertoli cells from different experimental groups. *n* = 3 technical repetitions. (F) Schematic model proposing whether ROS‐induced ZAKα phosphorylation through ribosome collisions in Sertoli cells. (G) Immunofluorescence staining for Mitosox (red) and DAPI (blue) in Sertoli cells from different experimental groups. *n* = 6. Scale bar = 10 μm. (H) Polysome profiling showed the change of global translation of different experimental groups in Sertoli cells. *n* = 3 technical repetitions. (I) Western blot analysis for puromycin incorporation in Sertoli cells from different experimental groups. *n* = 3 technical repetitions. (J) Polysome profiling of Sertoli cells in the presence of Mnase. The graph shows the absorbance at OD260 for Control (blue) and PA (red) samples and black arrow indicates a disome peak. *n* = 3 technical repetitions. Disomes are stable complexes formed by the physical stacking of two ribosomes on an mRNA, serving as a direct biochemical signature of ribosome collision. Therefore, an increase in disome abundance specifically reflects elevated collision events. (K) Western blot shows the level of ZAKα, p38, and p‐p38 in Sertoli cells from different treatment groups. *n* = 3 technical repetitions. (L) Western blot shows the level of ZAKα, p38, and p‐p38 in Sertoli cells from different experimental groups. *n* = 3 technical repetitions. Bars represent means ± SEM. Statistical significance was assessed using one‐way ANOVA followed by Tukey's post hoc tests or Kruskal–Wallis test with Dunn post hoc tests. *p < 0.05, **p < 0.01, ***p < 0.001, ****p < 0.0001.

To further explore the mechanism of ROS‐induced aging in Sertoli cells, we obtained primary Sertoli cells from old mice and young mice and performed RNA sequencing. The volcano plot revealed significant differential gene expression between aging and control groups, marked by numerous upregulated (pink) and downregulated (blue) genes (Figure 3A). GO enrichment analysis identified aging‐linked biological processes, with cytoplasmic translation emerging as a significantly enriched process, followed by mitochondrial respiratory chain assembly and oxidative phosphorylation, collectively reflecting critical transcriptional alterations associated with aging (Figure 3B). Cytoplasmic translation was significantly downregulated in aging, with a negative enrichment score (NES: −2.81) and high statistical significance (*p* value < 0.001), indicating reduced translational activity in aged Sertoli cells (Figure 3C). Guided by the RNA‐seq findings revealing downregulated translation machinery, we next sought to determine whether these transcriptomic changes resulted in functional deficits in protein synthesis. To experimentally validate bioinformatics results, polysome profiling and immunoblotting analyses using Puromycin were performed. Polysome profiling and immunoblotting analyses demonstrated diminished translational activity in aging and PA‐treated Sertoli cells, marked by reduced polysome/monosome ratios and lower Puro/ACTB expression levels, respectively (Figure 3D,E). According to Simon (Snieckute et al. 2023), H_2_O_2_‐induced ROS in U2OS cells can cause ribosome collisions, thereafter activating the ZAKα protein. Thus, we speculate whether the decline in protein synthesis in senescent Sertoli cells of the testes is similarly caused by ribosome collisions (Figure 3F). Therefore, we performed ROS detection and found that PA‐treated Sertoli cells significantly increased mitochondrial ROS, while NAC effectively scavenged mitochondrial ROS (Figure 3G). PA treatment impaired translational activity, while NAC co‐treatment partially reversed these effects, suggesting ROS contributes to PA‐induced translational suppression and NAC alleviates this damage through antioxidant activity (Figure 3H,I). Moreover, the polysome profiling results showed that PA treatment increased the disome peak compared to the control, suggesting that PA can actually induce ribosome collisions in Sertoli cells (Figure 3J). Furthermore, ribosome collisions occurred in aging and PA‐treated Sertoli cells, as indicated by increased levels of ZAKα and p‐p38. These increases were attenuated by NAC co‐treatment, suggesting that scavenging ROS reduced the occurrence of ribosome collisions (Figure 3K,L). These findings establish ROS as a driver of ribosome collisions in Sertoli cells, activating ZAKα‐p38 signaling to suppress global translation and accelerate senescence. Antioxidant intervention (NAC) mitigates collision burden, highlighting ROS clearance as a potential strategy to preserve translational activity and cellular homeostasis.

### 2.5 ROS Induces Ribosome Stalling and Collisions to Disrupt mRNA Translation

**FIGURE 4:**
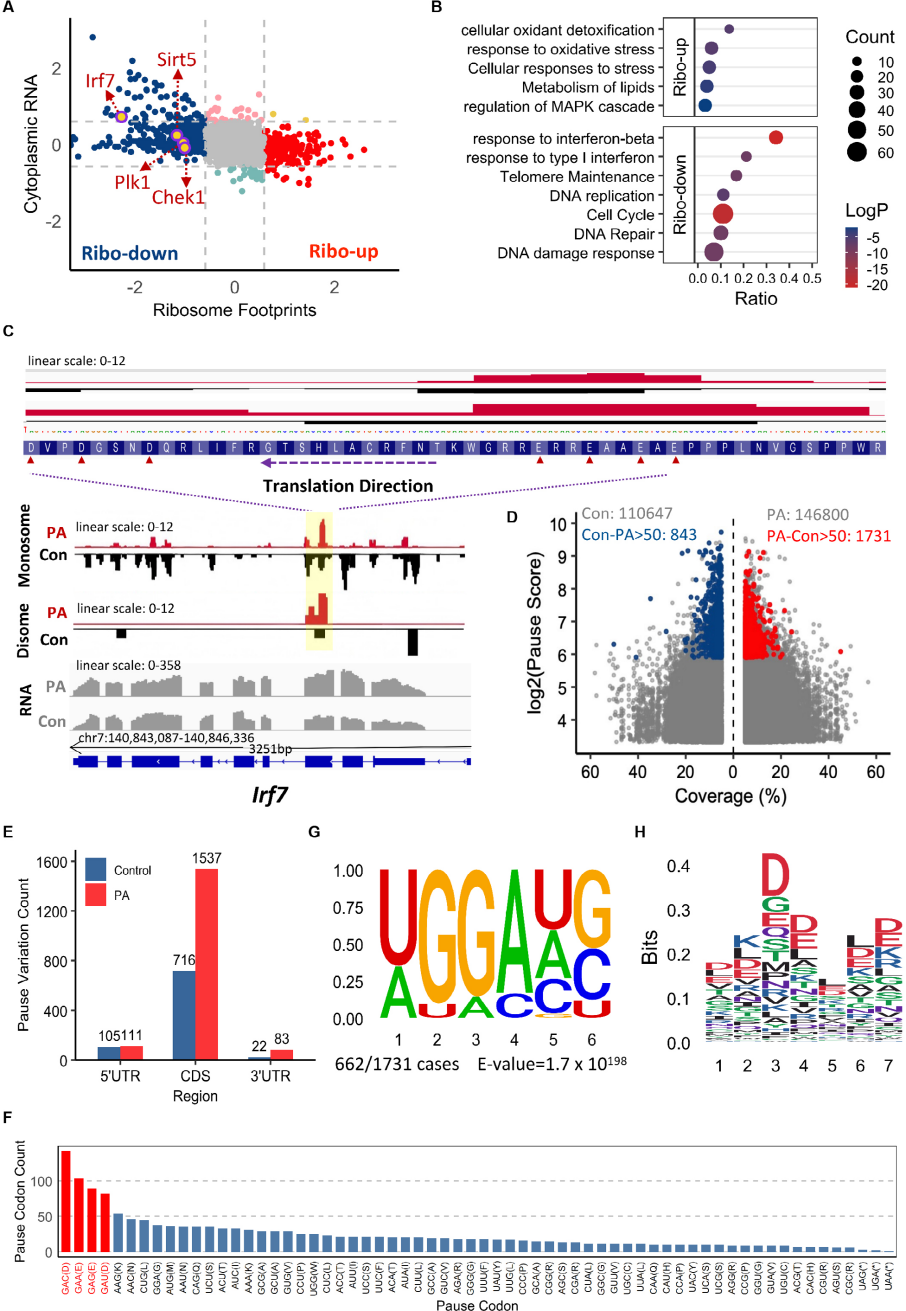
ROS induces ribosome stalling and collisions (A) Scatterplot comparing the fold changes of ribosomal footprints (X‐axis) and cytoplasmic RNA levels (Y‐axis), with downregulated genes (blue) and upregulated genes (red) identified by Monosome‐seq (Ribo‐seq) in PA‐treated cells versus controls. (B) Gene Ontology (GO) enrichment analysis of translationally up‐/down‐regulated mRNAs in response to PA treatment. (C) Track plot showing a marked reduction in translation efficiency, but not in RNA levels, of Irf7 in PA‐treated cells compared to control cells. Translation of Irf7 mRNA was specifically stalled at a region harboring D and E codons, which are indicated by red triangles. D: aspartate, E: glutamate. (D) Volcano plot compares pause scores and coverage (%) between PA‐treated and control cells. Sites with pause scores 50 points higher in the PA group (vs. control) are highlighted in red, while those with scores 50 points higher in the control group (vs. PA) are marked in blue. (E) The distribution plot of pause sites with pause scores greater than 50 in the PA group (vs. control) across different regions of the mRNA. (F) Statistics of codon frequencies at pause sites in the PA group with pause scores 50 points higher than the control group. The top four ranked codons are labeled in red. (G) Enrichment plots of sequence motif within 25 nucleotides surrounding pause sites (*n* = 1731 sites with pause score > 50 in PA group vs. control). (H) Peptide motif (7‐aa) around pause sites (*n* = 1537 sites with pause score > 50 in PA group vs. controls).

To elucidate the molecular mechanisms connecting the buildup of ROS with translational dysregulation during aging, we next performed monosome footprinting (Ribo‐seq) and disome footprinting (Disome‐seq) to comprehensively analyze the impact of ROS on mRNA translation (Figure S2A,B). Remarkably, a substantially greater change in translation efficiency was observed compared to mRNA levels. Monosome‐seq data revealed that 420 genes exhibited a significant increase in translation efficiency, while 648 genes showed a significant decrease in PA‐treated Sertoli cells relative to control cells (Figure 4A). Furthermore, translation initiation was markedly reduced in PA‐treated Sertoli cells compared to controls (Figure S2C). Codon occupancy analysis further demonstrated an increased ribosome frequency at stop codons and a decreased ribosome frequency at start codons (Figure S2D). Collectively, these results are consistent with the above finding that ROS robustly suppresses mRNA translation. Subsequently, Gene Ontology analysis was conducted to categorize mRNA translational changes. The results revealed that genes exhibiting increased translation efficiency were primarily associated with oxidative stress response and lipid metabolism. Conversely, genes with reduced translation efficiency were predominantly linked to aging‐related pathways, such as telomere maintenance (Lin and Epel 2022), DNA damage response (Ahmad et al. 2024), and cell cycle regulation (Gorgoulis et al. 2019) (Figure 4B). Consistent with the aging process, PA‐treated cells showed severely suppressed translation efficiency of SIRT5 (Zhao et al. 2025), Ilr7 (Jiang et al. 2024; Qin et al. 2022), Plk1 (Driscoll et al. 2014), and Chek1 (Bianco et al. 2019) mRNAs, which have been known to be directly or indirectly involved in aging (Figure 4A). Interestingly, we observed significant translational stalling of these transcripts in PA‐treated cells, accompanied by a pronounced collision at the same locus detected by Disome‐seq (Figure 4C and Figure S2E). More importantly, using Pausepred (Kumari et al. 2018), we detected a marked increase in ribosome pausing in the PA‐treated group compared to the control group, identifying 146,800 confidence pause sites (coverage > 5%, pause score > 10) in the PA group, in contrast to 110,647 sites in the control group (Figure 4D). Among the predicted pause sites, 1731 candidates in the PA group displayed a significant change in pause score, with a difference of at least 50 compared to the corresponding positions in the control alignments. Importantly, the genes prone to such translational pausing are primarily associated with the aging process (Figure S2F). Additionally, 1537 pause sites were located within the coding sequence (CDS) region (Figure 4E), with the predominant pausing codons at the A‐site being GAC (Aspartate/D), GAA (Glutamate/E), GAG (Glutamate/E), and GAU (Aspartate/D) (Figure 4F). To investigate potential regulatory motifs influencing age‐dependent ribosome pausing, we examined the 25 nucleotides surrounding the detected pause sites within the 1731 candidate sequence. Using Multiple EM for Motif Elicitation (MEME), we identified a highly significant GA‐rich motif present in 662 sites of these sequences (Figure 4G). Notably, there was a significant positional enrichment of certain amino acids, with aspartate and glutamate enriched around multiple ribosomal pause sites (Figure 4H). Taken together, these data offer further support for the role of ROS in inducing ribosome stalling and collisions, leading to disruptions in mRNA translation, particularly at GA‐rich sequences encoding aspartate and glutamate.

### 2.6 ZAKα Knockdown Attenuates PA‐Induced Sertoli Cells Senescence and Functional Impairment

**FIGURE 5:**
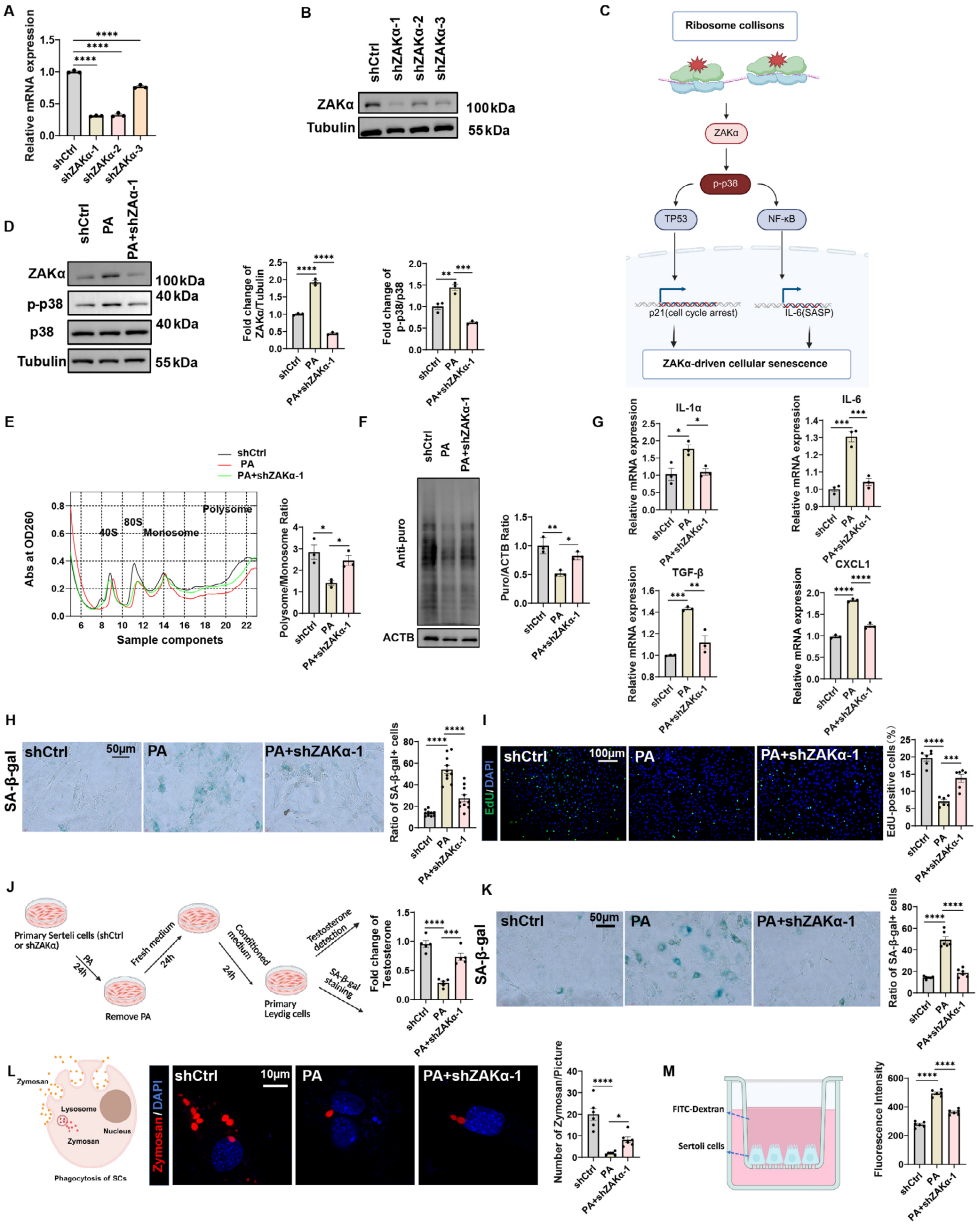
ZAKα knockdown rescued PA‐induced Sertoli cells senescence (A) relative mRNA expression levels of ZAKα in Sertoli cells transfected with different constructs (shCtrl: Negative control, shZAKα‐1, shZAKα‐2, and shZAKα‐3). (B) Western blot analysis to validate the knockdown efficiency of ZAKα in Sertoli cells. (C) Schematic model of ZAKα‐driven cellular senescence. (D) Western blot shows the level of ZAKα, p38, and p‐p38 in Sertoli cells from different treatment groups. *n* = 3 technical repetitions. (E) Polysome profiling showed the change of global translation of different experimental groups in Sertoli cells. *n* = 3 technical repetitions. (F) Western blot analysis for puromycin incorporation in Sertoli cells from different treatment groups. *n* = 3 technical repetitions. (G) Quantitative analysis of SASP‐related gene expression. Relative mRNA levels of IL‐1α, IL‐6, TGF‐β, and CXCL1 in shCtrl, PA, and PA + shZAKα‐1 groups. (H) SA‐β‐gal staining of Sertoli cells from different experimental groups. *n* = 10. Scale bar = 50 μm. (I) EdU incorporation assay in Sertoli cells from different treatment groups to measure cell proliferation. *n* = 6. Scale bar = 100 μm. (J, K) Schematic workflow for evaluating testosterone secretion and SA‐β‐gal in Leydig cells exposed to conditioned media from treated Sertoli cells (shCtrl, PA, PA + shZAKα‐1). Testosterone were quantified via ELISA. *n* = 5. SA‐β‐gal staining of Leydig cells exposed to conditioned media from treated Sertoli cells (shCtrl, PA, PA + shZAKα‐1). *n* = 6. Scale bar = 50 μm. (L) Schematic illustration of Zymosan particle phagocytosis by Sertoli cells. Representative fluorescence images showing Zymosan particles (red) and nuclei (blue) in different group. *n* = 6. Scale bar = 10 μm. (M) Schematic representation of Sertoli cells cultured with FITC‐Dextran. Bar graph quantifying FITC‐Dextran fluorescence intensity across experimental groups. *n* = 5. Bars represent means ± SEM. Statistical significance was assessed using one‐way ANOVA followed by Tukey's post hoc tests or Kruskal–Wallis test with Dunn post hoc tests. *p < 0.05, **p < 0.01, ***p < 0.001, ****p < 0.0001.

To investigate the functional role of ZAKα in aging‐associated phenotypes, we first validated its knockdown efficiency in Sertoli cells using three independent shRNA constructs (shZAKα‐1, shZAKα‐2, and shZAKα‐3). RT‐qPCR and immunoblotting analyses confirmed significant reductions in both mRNA and protein levels of ZAKα, with shZAKα‐1 exhibiting the highest silencing efficacy (Figure 5A,–B). Mechanistically, ribosome collisions activate ZAKα‐mediated p‐p38 signaling, triggering TP53/NF‐κB pathways that drive senescence via p21‐dependent cell cycle arrest and SASP‐associated IL‐6 production (Li et al. 2023; Zarubin and Han 2005) (Figure 5C). PA treatment increased ZAKα expression and p38 phosphorylation, effects markedly reduced by ZAKα knockdown (Figure 5D). Functionally, PA‐induced translational suppression (Figure 5E,F), SASP gene upregulation (IL‐1α, IL‐6, TGF‐β, and CXCL1; Figure 5G), senescence (elevated SA‐β‐gal+ cells; Figure 5H), and cell cycle arrest (reduced EdU+ cells; Figure 5I) were significantly attenuated by ZAKα knockdown. We also investigated the effects of Sertoli cell senescence on Leydig cell function. The results showed that conditioned media from senescent Sertoli cells impaired testosterone secretion in Leydig cells and promoted their senescence. However, shZAKα‐1 transfection reduced these adverse effects (Figure 5J,K). PA treatment impaired phagocytic function, while shZAKα‐1 transfection partially restored phagocytic activity, highlighting ZAKα's critical role in mediating PA‐induced phagocytic dysfunction (Figure 5L). Besides, PA treatment also significantly increased the concentration of FITC‐Dextran in the lower culture medium compared to the shCtrl group, indicating weakened intercellular connections. However, shZAKα‐1 transfection in PA‐treated Sertoli cells reduced the concentration of FITC‐Dextran in the lower culture medium (Figure 5M). Besides, consistent with the results from shZAKα‐1, the knockdown of either shZAKα‐2 or shZAKα‐3 also alleviated PA‐induced senescence in Sertoli cells (Figure S3). Collectively, these data establish ZAKα as a central mediator of PA‐induced Sertoli cell senescence, orchestrating ribosome collision signaling to impair translation, amplify SASP, and disrupt key physiological functions. Targeting ZAKα may represent a therapeutic strategy to counteract lipid stress‐driven testicular dysfunction.

### 2.7 The ZAKα Inhibitor Nilotinib Ameliorates the Testes of Aged Mice

**FIGURE 6:**
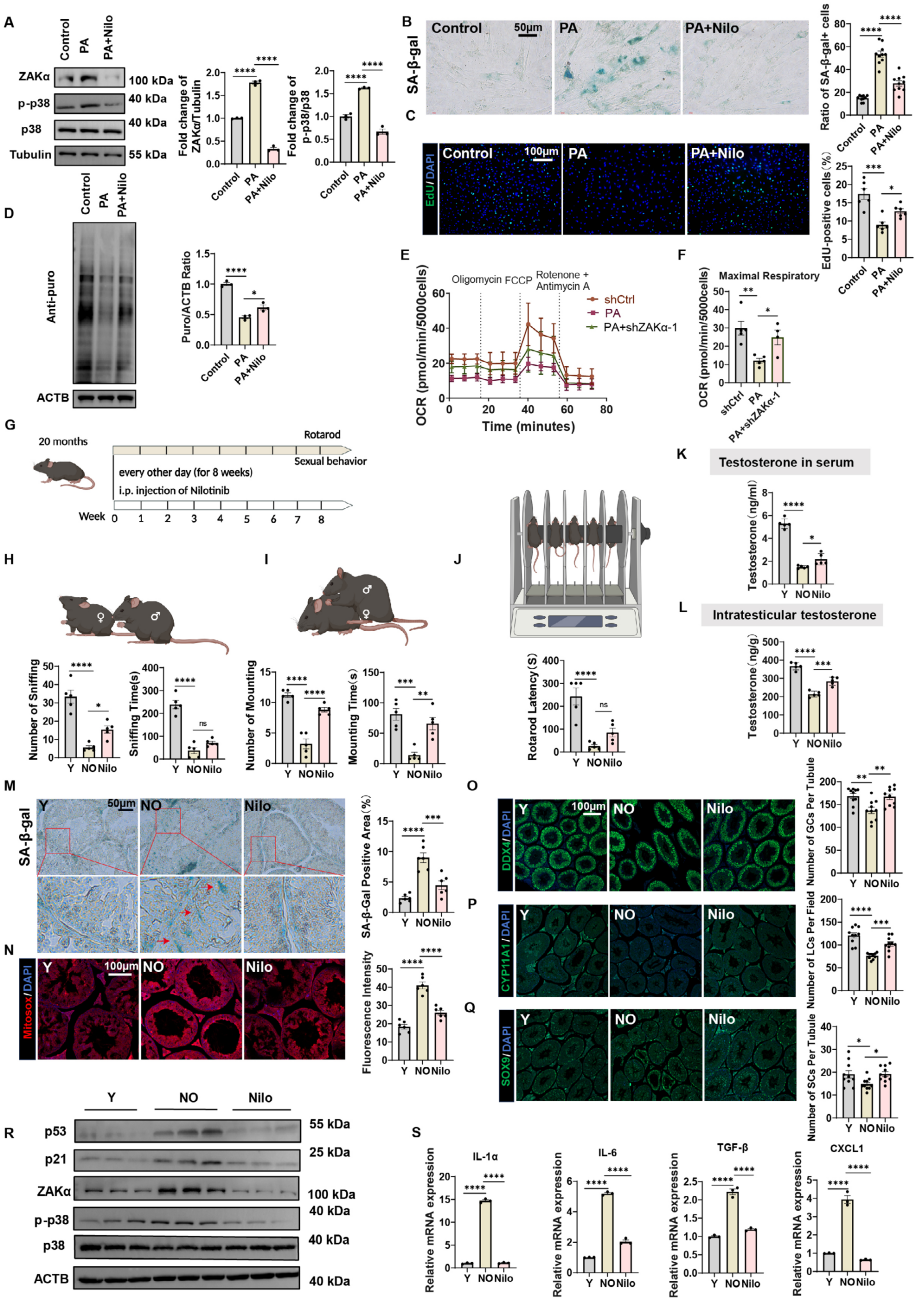
The ZAKα inhibitor nilotinib ameliorates the testes of aged Mice (A) Western blot shows the level of ZAKα, p38, and p‐p38 in Sertoli cells from different treatment groups. *n* = 3 technical repetitions. (B) SA‐β‐gal staining of Sertoli cells from different experimental. *n* = 6. Scale bar = 50 μm. (C) EdU incorporation assay in Sertoli cells from Control, PA, PA + Nilo groups to measure cell proliferation. *n* = 6. Scale bar = 100 μm. (D) Western blot analysis for puromycin incorporation in Sertoli cells from Control, PA, PA+Nilo groups. *n* = 3 technical repetitions. (E, F) Oxygen consumption rate (OCR) measurement of Sertoli cells from Control, PA, PA+Nilo groups. Maximal respiratory capacity of Sertoli cells from Control, PA, PA+Nilo groups, as determined by OCR. *n* = 4. (G) Experimental timeline for assessing the effects of Nilotinib on aging‐related behavioral decline in 20‐month‐old mice. Mice received intraperitoneal injections of Nilotinib every other day for 8 weeks, followed by Rotarod and sexual behavior tests at week 8. (H, I) Sniffing and mating behavior of mice with indicated treatment (Y, NO, Nilo). *n* = 5. (J) Rotarod analysis showing the stamina of mice with indicated treatment (Y, NO, Nilo). *n* = 5. (K, L) Intratesticular testosterone and Serum total testosterone concentration in the three experimental groups. *n* = 5. (M) Representative images of SA‐β‐gal staining of testicular tissue sections. Red arrows indicate SA‐β‐gal‐positive signals. Scale bar = 50 μm. (N) Immunofluorescence staining for Mitosox (red) and DAPI (blue) in testicular tissue sections. *n* = 6. Scale bar = 100 μm. (O–Q) Immunofluorescence staining of DDX4 (germ cell marker), CYP11A1 (LC marker), and SOX9 (SC marker) in testicular tissue sections. *n* = 10. Scale bar = 100 μm. (R) Western blot shows the level of ZAKα, p38, p‐p38, p53, and p21 in testicular tissue from different treatment groups. (S) Quantitative analysis of SASP‐related gene expression. Relative mRNA levels of IL‐1α, IL‐6, TGF‐β, and CXCL1 from different testicular tissue. Bars represent means ± SEM. Statistical significance was assessed using one‐way ANOVA followed by Tukey's post hoc tests or Kruskal–Wallis test with Dunn post hoc tests. *p < 0.05, **p < 0.01, ***p < 0.001, ****p < 0.0001.

To investigate the role of ZAKα inhibitor Nilotinib in vitro, we first treated Sertoli cells with PA and observed a significant upregulation of ZAKα expression and p‐p38 phosphorylation compared to controls. Co‐treatment with ZAKα inhibitor Nilotinib attenuated these effects (Figure 6A). PA treatment markedly increased the ratio of SA‐β‐gal+ Sertoli cells, which was reversed by Nilotinib (Figure 6B). Furthermore, PA impaired cell proliferation, as evidenced by reduced EdU+ cells, and suppressed translational activity, indicated by a decreased Puro/ACTB ratio. Both phenotypes were partially rescued by Nilotinib (Figure 6C,D). PA also induced mitochondrial dysfunction, while Nilotinib co‐treatment restored it (Figure 6E,F). These data suggest that Nilotinib ameliorates Sertoli cell senescence in vitro. To assess the physiological relevance of these findings in vivo, we administered Nilotinib (10 mg/kg, i.p.) every other day for 8 weeks to 20‐month‐old mice. Aged (NO group) and Nilotinib‐treated aged (Nilo group) mice underwent physiological evaluations (Figure 6G). Notably, NO group mice exhibited decreased testis‐to‐body weight ratios compared to young (Y group) controls, whereas Nilotinib treatment restored this ratio (Figure S4A,B). Sexual behaviors, assessed via sniffing and mounting tests, were impaired in NO mice but partially recovered in the Nilo group (Figure 6H,I). Rotarod test results showed that the NO group exhibited significantly reduced latency time compared to the Y group, indicating impaired motor function in aged mice. Nilotinib treatment showed a trend of improving latency time, though the difference was not statistically significant (Figure 6J). Both serum and intratesticular testosterone levels were significantly reduced in the NO group compared to the Y group, whereas treatment with Nilotinib partially restored testosterone levels (Figure 6K,L). NO group testes exhibited increased SA‐β‐gal staining compared to Y group, demonstrating heightened cellular senescence, which was attenuated by Nilotinib treatment (Figure 6M). Similarly, mitochondrial ROS levels were elevated in NO group versus Y group, while Nilotinib administration significantly reduced ROS (Figure 6N). Notably, Nilotinib preserved testicular cellular composition, rescuing populations of DDX4+ germ cells, SOX9+ Sertoli cells, and CYP11A1+ Leydig cells that were diminished in aged mice (Figure 6O–Q). Histological analysis revealed age‐associated pathologies in NO group testes, kidneys, livers, and muscles, including vacuolization and structural disorganization. These abnormalities were ameliorated by Nilotinib (Figure S4C). Ultrastructural analysis via TEM demonstrated mitochondrial swelling in NO group Sertoli cells, which was mitigated by Nilotinib (Figure S4D). NO group exhibited increased lipid deposition in Sertoli cells of testicular tissue compared to Y group, whereas Nilotinib treatment attenuated this age‐related lipid accumulation (Figure S4E). Cell cycle‐related proteins (p53 and p21), ZAKα and p‐p38 were elevated in aged testes but reduced by Nilotinib (Figure 6R). Moreover, the expression of SASP factors (IL‐1α, IL‐6, TGF‐β, and CXCL1) was similarly suppressed in Nilo group testes (Figure 6S). These findings collectively demonstrate that pharmacological inhibition of ZAKα by Nilotinib mitigates multiple hallmarks of aging, including cellular senescence, mitochondrial dysfunction, and tissue degeneration in aged mice.

## 3 Discussion

Our study establishes lipid droplet (LD)‐driven mitochondrial ROS as a pivotal mechanism underlying Sertoli cells' senescence and testicular aging, uncovering a previously unrecognized axis involving ribosome collisions and ZAKα activation in the reproductive system. These findings align with emerging evidence that metabolic lipid droplet overload disrupts organ function in aging tissues, such as brain, liver, heart, and kidney (Gluchowski et al. 2017; Han et al. 2023; Haney et al. 2024; Li, Munoz‐Mayorga, et al. 2024; Mitrofanova et al. 2023; Wu et al. 2021). The accumulation of LDs in Sertoli cells from LOH patients and PA‐treated models mirrors observations in neurodegenerative and hepatic disorders, where lipid peroxidation and mitochondrial ROS amplify cellular dysfunction (Mi et al. 2023). However, our work links LD‐associated oxidative stress to ribotoxic signaling in the context of reproductive aging, expanding the paradigm of redox biology beyond classical DNA or protein damage.

Central to this mechanism is the collapse of mitochondrial integrity triggered by LD overload. Sertoli cells, tasked with sustaining high‐energy spermatogenesis, rely heavily on oxidative phosphorylation, rendering them susceptible to lipid‐induced ETC disruptions. The downregulation of mitochondrial complex subunits and translation machinery, as revealed by RNA‐seq, suggests that ROS overproduction depletes cellular capacity to maintain proteostasis—a phenomenon previously documented in neurons (Xu et al. 2022). Notably, we demonstrate that ROS generated from compromised mitochondria directly provoke ribosome collisions, a novel finding in reproductive biology. Ribosome collisions, typically associated with translation elongation errors or nutrient deprivation, are increasingly recognized as universal stress sensors. Our data extend this concept to lipid‐induced redox stress, wherein ROS perturb ribosome function, creating a self‐amplifying loop of ribotoxic signaling.

The activation of ZAKα, a master regulator of ribotoxic stress responses, emerges as a critical checkpoint in this cascade. ZAKα's role in transmitting collision signals to p38 pathways has been characterized in viral infection and UVB‐induced damage, but its involvement in aging‐associated senescence is unexplored (Karasik et al. 2024; Robinson et al. 2022; Vind et al. 2024). Our observation that ZAKα inhibition rescues Sertoli cells' function underscores its dual role as both a sensor and effector of redox‐metabolic imbalance. This aligns with recent studies showing that ZAKα ablation extends lifespan and mitigates hepatic fibrosis, suggesting evolutionary conservation of its pro‐aging functions (Rodríguez‐Ruiz et al. 2023; Snieckute et al. 2023). Importantly, the reversibility of senescence phenotypes upon ZAKα blockade highlights the dynamic nature of redox‐proteostatic dysregulation in testicular aging, challenging the notion of senescence as an irreversible endpoint.

While our findings illuminate a LD‐ROS‐ribosome collisions axis in aged Sertoli cells, several questions still remain. First, the precise molecular link between mitochondrial ROS and ribosomal stalling warrants further investigation. ROS may oxidize rRNA or ribosomal proteins, as suggested in neurodegenerative models, or deplete tRNA pools via oxidative damage—a hypothesis supported by the downregulation of ribosome biogenesis genes in our RNA‐seq data. Second, the crosstalk between ZAKα and other redox‐sensitive pathways in Sertoli cells remains to be mapped. Finally, while our PA model recapitulates key features of human LOH, age‐related testicular aging likely involves cumulative damage from multiple lipids and inflammatory mediators, necessitating studies in physiologically aged models.

In conclusion, we propose a paradigm in which LD accumulation acts as a metabolic “first hit” in Sertoli cells, sensitizing them to mitochondrial ROS‐mediated ribosome collisions, while ZAKα activation serves as the “second hit” that locks cells into senescence. This redox‐metabolic axis not only deepens our understanding of testicular aging but also provides a framework for targeting ribotoxic stress pathways in other age‐related disorders characterized by proteostatic collapse.

## 4 Materials and Methods

### 4.1 Animals

Naturally old mice and young mice with a C57BL/6 background were purchased from Guangdong Jinzhihe Biotechnology Co. Ltd. All animal experiments received approval from the Animal Ethics Committee of the Fifth Affiliated Hospital of Sun Yat‐sen University (Approval No. 00359) and were conducted according to the NIH Guide for the Care. Obese old (OO) mice were developed by exposing mice to an HFD (60 kcal% fat, Dyets, HF60) at 16 months of age for 4 months, while naturally old (NO) and young mice were fed with an ND (10 kcal% fat) food throughout the study period.

### 4.2 Mating Behavior Analysis

Female mice received intraperitoneal injections of estradiol (20 μg/mouse in 0.1 mL corn oil) 48 h before testing and progesterone (500 μg/mouse in 0.1 mL corn oil) 4 h pre‐test. Vaginal smears verified estrous stage prior to behavioral observations. For mating trials, male mice were acclimated to the test cage before introducing estrous females under dim light. Interactions were video‐recorded for 30 min immediately after introduction. Male sexual behavior was categorized as: Sniffing: Investigation of the female's genital/perianal area ending when disengaging. Mounting: Climbing onto the female with pelvic thrusts ending upon dismount. Frequency and duration of both behaviors were quantified over 30 min.

### 4.3 Rotarod Performance Assessment

Mice underwent habituation with seven stationary‐rod trials. During testing, animals were placed on an accelerating rotarod (5–20 rpm over 300 s). Rotational latency was recorded automatically via integrated sensors. Each mouse completed three trials at 20‐min intervals to minimize fatigue.

### 4.4 Primary Culture of Mouse Sertoli and Leydig Cells

Primary Sertoli and Leydig cells were isolated from testes of 8‐week‐old male C57 mice. After euthanasia, testes were decapsulated and seminiferous tubules digested in Enzyme I (2 mg/mL type IV collagenase and 10 mg/mL DNase I; 37°C, 15 min, gentle agitation every 5 min). Following gradient centrifugation, Leydig cells (supernatant) were isolated via NGFR‐based FACS (Invitrogen#12‐9400‐42, ME20.4). The pellet was digested in Enzyme II (4 mg/mL type IV collagenase, 2.5 mg/mL hyaluronidase, 2 mg/mL trypsin, and 10 mg/mL DNase I; identical conditions), filtered through 70 μm mesh, and centrifuged to pellet Sertoli cells. After 24‐h culture, both cell types underwent PBS washing to remove residual germ cells, with identity confirmed by immunofluorescence.

### 4.5 Senescence Induction of Primary Sertoli Cells

Primary Sertoli cells underwent senescence induction via six methods: Debris: 293T cell debris (5:1 debris: Sertoli ratio) in serum‐free medium for 24 h. PA: 150 μM palmitic acid in complete medium for 24 h. H_2_O_2_: 100 μM H_2_O_2_ in complete medium for 2 h → PBS wash → complete medium for 22 h. UV: 50 mJ/cm^2^ UV‐B irradiation (3 min) → 24 h culture. Rep: Serial passaging until proliferative arrest. Aging: Primary cells from 20‐month‐old testes.

### 4.6 Senescence Associated β‐Galactosidase (SA‐β‐Gal) Staining

For SA‐β‐gal staining of cultured cells and testicular tissue sections, the cellular β‐galactosidase staining kit (Beyotime, C0602) was used according to the manufacturer's instructions. Sections or cells were covered with staining solution and incubated at 37°C overnight. Sections were imaged under a microscope, and the percentage of SA‐β‐gal positive cells or tissue was calculated using ImageJ software.

### 4.7 Oil Red O Staining

Lipid accumulation was assessed by Oil Red O staining in cultured cells and frozen sections. After fixation in 4% PFA (15 min) and PBS rinsing, samples were equilibrated in 60% isopropanol, then stained with 0.5% Oil Red O (Beyotime C0158S) for 15 min at RT. Unbound dye was removed by gentle distilled water washes. Nuclei were counterstained with hematoxylin (3 min) before microscopic imaging.

### 4.8 Western Blot

Testicular tissues and primary Sertoli cells were homogenized in RIPA lysis buffer (Beyotime, P0013B) supplemented with protease and phosphatase inhibitors (Roche, 10390845). Protein concentrations were determined via BCA assay (Beyotime, P0010S). Equal amounts of protein (30 μg) were separated by SDS‐PAGE (4%–20% gradient gels, Bio‐Rad, 4561094) and transferred to PVDF membranes (Millipore, IPFL00010). Membranes were blocked with 5% nonfat milk in TBST and incubated overnight at 4°C with primary antibodies. After TBST washes, membranes were probed with HRP‐conjugated secondary antibodies for 1 h at room temperature. Signals were visualized using ECL Prime (Bio‐Rad, 1705062). Antibody specifications are detailed in Table S2.

### 4.9 Quantitative Real‐Time Reverse Transcription Polymerase Chain Reaction (RT‐qPCR)

Total RNA was isolated from mouse testicular tissues and primary Sertoli cells using TRIzol reagent (Thermo Fisher, 15596026), followed by DNase I treatment (Qiagen, 79254) to eliminate genomic DNA. Reverse transcription was performed with 1 μg RNA using the HiScript III RT SuperMix for qPCR (+gDNA wiper) (Vazyme, R323‐01). RT‐qPCR amplification was carried out in triplicate using ChamQ SYBR qPCR Master Mix (Vazyme, Q311‐02). Primer sequences for SASP associated genes (IL‐1α, IL‐6, TGF‐β, and CXCL1) and the housekeeping gene ACTB were designed using Primer Bank. Primer sequences are detailed in Table S1.

### 4.10 RNA Isolation and Sequencing of Primary Testicular Sertoli Cells

Primary Sertoli cells in 6‐well plates were lysed with TRIzol (Thermo Fisher 15596026) for RNA extraction. Lysates underwent chloroform phase‐separation/isopropanol precipitation, DNase I treatment (Qiagen 79254), and RNeasy Mini Kit purification (Qiagen 74104). RNA quality was verified (RIN > 8.0; A₂₆₀/A₂₈₀: 1.9–2.1) using Agilent 2100 Bioanalyzer/Qubit 4.0 (Thermo Fisher Q33238). Strand‐specific mRNA sequencing (≥ 1 μg RNA/replicate) was performed by Novogene on Illumina NovaSeq 6000 (PE150), with raw data quality controlled and adapter trimmed pre‐analysis.

### 4.11 Ribo‐Seq and Disome‐Seq Library Preparation in Testicular Primary Sertoli Cells

For Ribo‐seq and Disome‐seq analysis, primary Sertoli cells were cultured in 15‐cm plates to approximately 80%–90% confluence. Cells were treated with 100 μg/mL cycloheximide for 8 min at 37°C, then lysed on ice for 10 min in a buffer containing 20 mM Tris–HCl (pH 7.5), 150 mM NaCl, 5 mM MgCl2, 1 mM DTT, 100 μg/mL cycloheximide, and 1% Triton X‐100. The lysate was clarified by centrifugation at 12,000 × g for 10 min at 4°C. Ribosome‐protected fragments (RPFs) were isolated using MicroSpin S‐400 columns (Cytiva, 27514001) and purified with the Zymo Research RNA Clean & Concentrator‐25 kit (Zymo, R1003). The RPFs were size‐selected on a 15% polyacrylamide TBE–urea gel, with fragments between 25 and 35 nucleotides (Ribo‐seq), 50 and 80 nucleotides (Disome‐seq) excised and RNA recovered using the ZR small‐RNA PAGE Recovery Kit (Zymo, R1070). Purified RNA was treated with PNK, and libraries were prepared using the VAHTSTM Small RNA Library Prep Kit for Illumina (NR811‐01).

### 4.12 Pre‐Processing of Sequencing Data

Monosome‐seq (Ribo‐seq), Disome‐seq, and RNA‐seq data were pre‐processed using established methods as previously described (Chothani et al. 2022). Briefly, adapters and low‐quality bases were trimmed using TrimGalore (v0.6.7). The clean reads were initially aligned to rRNA sequences from the SILVA rRNA database (release 138) (Quast et al. 2013) and repetitive sequences from RepBase (v27.04) (Bao et al. 2015) using Bowtie (v1.3.1) (Langmead et al. 2009) with the parameters ‐v 1–best. Unaligned reads were then demultiplexed for subsequent downstream analysis.

### 4.13 Monosome‐Seq (Ribo‐Seq), Disome‐Seq, and RNA‐Seq Data Analysis

The processed reads were aligned to the reference genome (GRCm39) using STAR (v2.7.10a) (Dobin et al. 2013) with a maximum allowance of two mismatches. Raw read counts were quantified using StringTie (v2.1.7) (Pertea et al. 2015). Both Monosome‐seq (Ribo‐seq), Disome‐seq, and RNA‐seq read counts were normalized to TPM (transcripts per million). Quality control was performed using the Ribo‐Toolkit (Liu et al. 2020). Genes with sufficient expression level (Ribo TPM > 1 and RNA TPM > 1) were subjected to further analysis. Translation efficiency (TE) and the differentially‐TE genes (DTEGs) were calculated using RiboDiff (v0.2.2) (Zhong et al. 2017). GO term analysis was performed by Metascape (https://metascape.org/gp/index.html). Ribosome pauses and Pause‐codon were identified by PausePred (Kumari et al. 2018). Sequence motifs were analyzed with MEME (https://meme‐suite.org/meme/tools/meme). R was used for data presentation.

### 4.14 Polysome Profiling

For polysome profiling, primary Sertoli cells were cultured in 15‐cm plates to approximately 80%–90% confluence. Cells were treated with 100 μg/mL cycloheximide for 8 min at 37°C, then lysed on ice for 10 min in a buffer containing 20 mM Tris–HCl (pH 7.5), 150 mM NaCl, 5 mM MgCl2, 1 mM DTT, 100 μg/mL cycloheximide, and 1% Triton X‐100. The lysate was clarified by centrifugation at 12,000 × g for 10 min at 4°C. Ribosome‐containing supernatants with equal A260 were loaded onto a 10%–50% sucrose gradient and centrifuged in an SW 41 Ti rotor (Beckman) at 4°C for 3 h at 35,000 rpm. Polysome profiles were generated and analyzed using the Gradient Station system.

### 4.15 Testosterone Measurement

Testosterone levels in testicular homogenates and Leydig cell supernatants were measured using an ELISA kit (ABclonal RK00724). Testes were homogenized in ice‐cold PBS (pH 7.4, 0.1% Triton X‐100, 1× protease inhibitor) using a Dounce homogenizer, then centrifuged (12,000 × g, 15 min, 4°C). Leydig cell media were cleared by centrifugation (3000 × g, 10 min). Samples (50 μL)—standards, 1:10 diluted homogenates (testicular tissue), or supernatants—were added to pre‐coated wells with 50 μL HRP‐conjugated detection antibody. After 1 h incubation (37°C), plates were washed 5×, developed with 100 μL TMB substrate, stopped, and measured at 450 nm. Curve validity was confirmed by verifying the absence of systematic bias in the model fit. Furthermore, functional accuracy required all back‐calculated standard concentrations to fall within ±15% of their nominal values.

### 4.16 Seahorse

To assess mitochondrial respiratory function, we used the XF96 Extracellular Flux Analyzer (Agilent) to measure the oxygen consumption rate (OCR) in primary Sertoli cells. Briefly, 5 × 10^3^ cells were seeded per well in XF96 cell culture microplates and incubated overnight at 37°C with 5% CO_2_. The next day, cells were washed three times with XF assay medium and incubated in a CO_2_‐free environment for 1 h. OCR was measured sequentially upon injection of oligomycin (1 μM), FCCP (2 μM), and rotenone/antimycin A (0.5 μM). Data were analyzed using the XF96 software.

### 4.17 Phagocytosis Assay

pHrodo Red Zymosan A bioparticles (Invitrogen, P35364) were resuspended at 0.5 mg/mL in D/F‐12 medium containing FBS and penicillin–streptomycin, then sonicated to ensure homogeneous dispersion. The culture medium from Sertoli cells was replaced with this bioparticle suspension, followed by incubation at 37°C for 24 h to allow maximal uptake. After fixation, cells were kept in the dark until analysis via fluorescence microscopy using the appropriate channel (Ex/Em: 560/585 nm).

### 4.18 Puromycin Incorporation Assays

Primary Sertoli cells were treated as indicated, then exposed to medium containing puromycin (10 μg/mL) for 10 min. After treatment, cells were washed with ice‐cold PBS and lysed immediately. The incorporation of puromycin into nascent polypeptide chains was detected by Western blot using the anti‐puromycin antibody.

### 4.19 HE and Masson's Trichrome Staining

Testicular specimens were collected, rinsed in PBS to remove residual blood, and fixed in 4% paraformaldehyde at 4°C for 24 h. Fixed tissues were embedded sequentially in paraffin blocks and OCT matrix, then sectioned at 10 μm. For Masson's trichrome staining, sections underwent sequential treatment with: Weigert's hematoxylin (nuclei), ponceau‐acid fuchsin solution (cytoplasm), and aniline blue (collagen). For HE staining, sections were immersed in Harris hematoxylin (nuclear morphology) followed by eosin Y counterstain (cytoplasm/extracellular matrix). Finally, all stained sections were dehydrated through a graded ethanol series and mounted in neutral resin for microscopic analysis.

### 4.20 Transmission Electron Microscopy of Testes

Testis tissues were dissected, trimmed of fat and vessels, and fixed in EM fixative for 24 h. Gradient dehydration was performed using acetone (30%–100%), followed by two 100% acetone washes (20 min total). For embedding, tissues were infiltrated in acetone‐embedding agent (3:1) at 37°C for 1 h, then pure embedding agent overnight. Samples were molded and baked at 60°C. Polymerization occurred at 60°C for 48 h. Ultrathin sections (70–90 nm) were cut and collected on copper grids. Staining included uranyl acetate (8–15 min) and lead citrate (5–10 min). Sections were imaged via TEM for morphological analysis.

### 4.21 Immunofluorescence (IF) Staining of Testicular Tissues

Testicular tissues were fixed in 4% paraformaldehyde (PFA) overnight at 4°C, dehydrated in 30% sucrose, and embedded in OCT. Cryosections were permeabilized with 0.3% Triton X‐100 in PBS and subjected to antigen retrieval using sodium citrate buffer (pH 6.0, 95°C, 15 min). Sections were blocked with 5% BSA/0.1% Tween‐20 in PBS for 1 h and incubated overnight at 4°C with primary antibodies. After PBST washes, sections were probed with Alexa Fluor‐conjugated secondary antibodies for 1 h at room temperature. Nuclei were counterstained with DAPI. Images were acquired using a Zeiss LSM 980 confocal microscope. Fluorescence intensity was quantified using ImageJ. Antibody specifications are detailed in Table S2.

### 4.22 EdU Assay

The 5‐ethynyl‐2′‐deoxyuridine (EdU) assay was conducted to assess cell proliferation. Cultured Sertoli cells were incubated with 10 μM EdU (Beyotime, C10071S) for 2 h, followed by fixation and permeabilization. Click reaction with Alexa Fluor 594‐azide was performed to detect EdU incorporation, while nuclei were counterstained with Hoechst 33342. Fluorescent signals were captured using confocal microscopy, and EdU‐positive cells were quantified via ImageJ.

### 4.23 JC‐1 Assay

The JC‐1 (5,5′,6,6′‐tetrachloro‐1,1′,3,3′‐tetraethylbenzimidazolylcarbocyanine iodide) assay was performed to evaluate mitochondrial depolarization. Sertoli Cells were incubated with JC‐1 dye (Solarbo, M8650) at 37°C for 30 min, followed by PBS washing. Fluorescent signals were captured by confocal microscopy. Red/green fluorescence ratios were quantified using ImageJ to assess mitochondrial integrity.

### 4.24 ROS Detection

Testicular tissue slices or cultured Sertoli cells for total ROS detection were incubated with 2.5 μM CellROX (Thermo, C10448) at 37°C for 30 min, followed by PBS washing. Testicular tissue slices or cultured Sertoli cells for mitochondrial ROS detection were incubated with 5 μM Mitosox (Thermo, M36008) at 37°C for 10 min, followed by PBS washing. Fluorescence intensity (Ex/Em: 488/525 nm) was quantified via confocal microscopy.

### 4.25 Lentiviral shZAKα Delivery and Transfection

Three shRNAs targeting ZAKα (shZAKα) were constructed in a lentiviral vector (pLKO.1) with a puromycin resistance marker (Tsingke Biotechnology). HEK293T cells were co‐transfected with packaging plasmids (psPAX2/pMD2.G) using PEI reagent. Viral supernatants were harvested at 48 h post‐transfection, concentrated via ultracentrifugation. Sertoli cells were infected with shZAKα lentivirus in polybrene‐containing medium (8 μg/mL), followed by puromycin selection (2 μg/mL, 72 h). ZAKα knockdown efficiency was validated by RT‐qPCR and Western blot.

### 4.26 The Permeability Assay of Sertoli Cells

The permeability assay of Sertoli cells was performed using a 24‐well Transwell chamber with a pore size of 0.4 μm (Corning). Fluorescein isothiocyanate (FITC)‐labeled dextran (0.5 mg/mL) (Merck) was introduced into the upper chamber and incubated at 37°C for 2 h. The fluorescence intensity of the lower chamber solution (Ex/Em: 492/530 nm) was assessed using a high‐throughput multimode detection system.

### 4.27 Statistical Analysis

Quantifications of fluorescence images and immunoblotting were performed using ImageJ (version 1.8.0). All data analysis was performed using GraphPad Prism (version 8.0). Data were showed as means ± SEM. Statistical significance was assessed using one‐way ANOVA followed by Tukey's post hoc tests or Kruskal–Wallis test with Dunn post hoc tests. *p < 0.05, **p < 0.01, ***p < 0.001, ****p < 0.0001.

## Author Contributions

Yanghua Xu: Investigation, methodology, visualization, writing – review and editing. Xiaoyan Shi: Methodology and writing. Yinghao Yin, Xiaopeng Tang, Biao Liu, Yuzhuo Chen, Zhitao Han, Jiarong Xu, Zitaiyu Li, Xiaoping Zheng, and Wanyi Xia: Methodology and visualization. Xiaoli Tan: Investigation and methodology. Ningjing Ou: Visualization. Hongshan Xie: Investigation and data curation. Hongji Hu: Formal analysis, resources, and supervision. Wenjing Wang: Formal analysis, resources and supervision. Hao Chen and Yuxin Tang: Project administration and funding acquisition. Liangyu Zhao: Conceptualization, project administration, funding acquisition, and Writing – review and editing.

## Funding

This work was supported byNational Key Research and Development Program of China 2022YFC2702705 and National Natural Science Foundation of China (82201756 to L.Z., 82571842 to Y.T. and 82401889 to Y.Y.).

## Conflicts of Interest

The authors declare no conflicts of interest.

## Supporting information


**Table S1:** Primer sequences used for real‐time quantitative PCR analyses.
**Table S2:** Antibody used for immunoblotting and immunofluorescence staining.
**Figure S1:** Obesity exacerbates testicular aging in vivo and in vitro (A) Immunofluorescence staining for DAPI (blue) and CYP11A1 (yellow) in testicular tissue sections. *n* = 10. Scale bar = 50 μm. (B) Immunofluorescence staining for VIM (red) and DDX4 (green) in testicular tissue sections. *n* = 10. Scale bar = 100 μm. (C) Immunofluorescence staining for DAPI (blue) and SOX9 (green) in testicular tissue sections. *n* = 10. Scale bar = 100 μm. (D, E) Representative images of HE and Masson staining of muscle, liver, kidney tissue sections. Scale bar = 100 μm. (F) Representative images of testicular tissue sections stained with Oil Red O to visualize lipid droplets. Red arrows indicate lipid droplets. Scale bar = 50 μm. (G) Identification and purity assessment of Sertoli cells. Representative images show co‐localization of markers GATA4 (red) and SOX9 (green) in Sertoli cells. Negative controls are included. Scale bar = 20 μm. (H) Representative immunofluorescence images of CYP11A1 (green) in Leydig cells stained with DAPI (blue). The negative control was processed without the primary antibody. Scale bar = 50 μm. Bars represent means ± SEM. Statistical significance was assessed using one‐way ANOVA followed by Tukey's post hoc tests or Kruskal‐Wallis test with Dunn post hoc tests. *p < 0.05, **p < 0.01, ***p < 0.001, ****p < 0.0001.
**Figure S2:** ROS induces ribosome stalling and collisions (A) Schematic diagram of the sequencing strategy. Monosome‐seq (Ribo‐seq) and Disome‐seq were conducted simultaneously in a single experiment, with monosome (~30 nt) and disome (~60 nt) footprints being isolated separately for sequencing analysis. (B) Quality control of Monosome‐seq data in control (top) and PA treated (bottom) cells. (C) Average ribosome occupancy across different mRNA regions in the PA group (red line) and the control group (black line). (D) Ribosome occupancy at individual codons in P‐sites (top) and A‐sites (bottom). The three stop codons (showing significant increases) and the start codon (displaying a decrease) are highlighted in red. (E) Track plot showing a significant decrease in the translation efficiency of Plk1 (top) and Chek1 (bottom) in PA‐treated cells compared with control cells. Translation of Plk1 and Chek1 mRNAs was specifically arrested at regions containing D and E codons, which are indicated by red triangles. D: aspartate, E: glutamate. (F) Gene Ontology analysis of reactive pathway enrichment using the genes with high pause sites in PA treatment.
**Figure S3:** ZAKα Knockdown Rescued PA‐Induced Sertoli Cells Senescence (A) Western blot shows the level of ZAKα, p38, and p‐p38 in Sertoli cells from different treatment groups. (B) Western blot analysis for puromycin incorporation in Sertoli cells from different treatment groups. (C) SA‐β‐gal staining of Sertoli cells from different experimental groups. *n* = 10. Scale bar = 50 μm. (D) EdU incorporation assay in Sertoli cells from different treatment groups to measure cell proliferation. *n* = 6. Scale bar = 50 μm. (E) Representative fluorescence images showing Zymosan particles (red) and nuclei (blue) in different group. *n* = 6. Scale bar = 10 μm. (F) Schematic representation of Sertoli cells cultured with FITC‐Dextran. Bar graph quantifying FITC‐Dextran fluorescence intensity across experimental groups. *n* = 6. (G‐I) Schematic workflow for evaluating testosterone secretion and SA‐β‐gal in Leydig cells exposed to conditioned media from treated Sertoli cells. Testosterone were quantified via ELISA. *n* = 5. SA‐β‐gal staining of Leydig cells exposed to conditioned media from treated Sertoli cells. *n* = 10. Scale bar = 50 μm. Bars represent means ± SEM. Statistical significance was assessed using one‐way ANOVA followed by Tukey's post hoc tests or Kruskal–Wallis test with Dunn post hoc tests. *p < 0.05, **p < 0.01, ***p < 0.001, ****p < 0.0001.
**Figure S4:** The ZAKα inhibitor nilotinib ameliorates the testes of aged mice (A, B) Representative images of mice and testis in the three experimental groups: Y (young), NO (naturally old), and Nilo (naturally old + Nilotinib). Testis‐to‐body weight ratio across experimental groups. *n* = 5. (C) Representative images of HE staining from testis, kidney, liver and muscle. Red arrows indicate disorganized tubules. Scale bar = 50 μm. (D) Representative images show mitochondria in Sertoli cells from mice using transmission electron microscopy (TEM). Red arrows indicate mitochondrial swelling. Scale bars = 5 μm. (E) Representative images of testicular tissue sections stained with Oil Red O to visualize lipid content. Red arrows indicate lipid droplets. Scale bar = 50 μm. Bars represent means ± SEM. Statistical significance was assessed using one‐way ANOVA followed by Tukey's post hoc tests or Kruskal–Wallis test with Dunn post hoc tests. *p < 0.05, **p < 0.01, ***p < 0.001, ****p < 0.0001.

**Appendix S2:** acel70359‐sup‐0002‐AppendixS2.docx.

## Data Availability

The RNA‐seq data and translatome profiling data have been deposited in the Sequence Read Archive (SRA). Accession to cite for these SRA data: PRJNA1370450 and PRJNA1370496. For further information, resources, and reagents, please contact the Lead Contact, Liangyu Zhao (zhaoly37@mail.sysu.edu.cn).
