# Supplementary material for: Lipid Droplet‐Driven Ribosome Collisions Trigger ZAKα‐p38 Signaling to Accelerate Testicular Aging
Source: Aging Cell. 2026 Jan 2;25(1):e70359. doi: 10.1111/acel.70359 (PMC12759184; doi:10.1111/acel.70359)
Supplement: Supplementary file 1 — Table S1: Primer sequences used for real‐time quantitative PCR analyses. Table S2: Antibody used for immunoblotting and immunofluorescence staining. Figure S1: Obesity exacerbates testicular aging in vivo and in vitro (A) Immunofluorescence staining for DAPI (blue) and CYP11A1 (yellow) in testicular tissue sections. n = 10. Scale bar = 50 μm. (B) Immunofluorescence staining for VIM (red) and DDX4 (green) in testicular tissue sections. n = 10. Scale bar = 100 μm. (C) Immunofluorescence staining for DAPI (blue) and SOX9 (green) in testicular tissue sections. n = 10. Scale bar = 100 μm. (D, E) Representative images of HE and Masson staining of muscle, liver, kidney tissue sections. Scale bar = 100 μm. (F) Representative images of testicular tissue sections stained with Oil Red O to visualize lipid droplets. Red arrows indicate lipid droplets. Scale bar = 50 μm. (G) Identification and purity assessment of Sertoli cells. Representative images show co‐localization of markers GATA4 (red) and SOX9 (green) in Sertoli cells. Negative controls are included. Scale bar = 20 μm. (H) Representative immunofluorescence images of CYP11A1 (green) in Leydig cells stained with DAPI (blue). The negative control was processed without the primary antibody. Scale bar = 50 μm. Bars represent means ± SEM. Statistical significance was assessed using one‐way ANOVA followed by Tukey's post hoc tests or Kruskal‐Wallis test with Dunn post hoc tests. *p < 0.05, **p < 0.01, ***p < 0.001, ****p < 0.0001. Figure S2: ROS induces ribosome stalling and collisions (A) Schematic diagram of the sequencing strategy. Monosome‐seq (Ribo‐seq) and Disome‐seq were conducted simultaneously in a single experiment, with monosome (~30 nt) and disome (~60 nt) footprints being isolated separately for sequencing analysis. (B) Quality control of Monosome‐seq data in control (top) and PA treated (bottom) cells. (C) Average ribosome occupancy across different mRNA regions in the PA group (red line) and the control group (b [file ACEL-25-e70359-s001.zip › Supinfo.docx]

**Table S1. Primer sequences used for real-time quantitative PCR analyses**

| Target gene | Primer forward | Primer reverse |
| --- | --- | --- |
| ACTB | GGCTGTATTCCCCTCCATCG | CCAGTTGGTAACAATGCCATGT |
| IL-1α | AGTATCAGCAACGTCAAGCAA | TCCAGATCATGGGTTATGGACTG |
| IL-6 | CTGCAAGAGACTTCCATCCAG | AGTGGTATAGACAGGTCTGTTGG |
| TGF-β | CCACCTGCAAGACCATCGAC | CTGGCGAGCCTTAGTTTGGAC |
| CXCL1 | ACTGCACCCAAACCGAAGTC | TGGGGACACCTTTTAGCATCTT |
| MAP3K20 | TCCGGGAGCCAGATTTTGTG | TCCTCCACCGCAGTTTTCAA |

**Table S2. Antibody used for immunoblotting and immunofluorescence staining**

| Antibody | Source | Identifier |
| --- | --- | --- |
| Puromycin Rabbit pAb | ABclonal | A21205 |
| Anti-MLTK Antibody | HUABIO | ER63976 |
| ZAK Polyclonal antibody | Proteintech | 28761-1-AP |
| p21 Polyclonal antibody | Proteintech | 28248-1-AP |
| p53 Monoclonal antibody | Proteintech | 60283-2-lg |
| CDKN2A/p16 | Santa-Cruz | sc-1661 |
| p38 MAPK Antibody | CST | #9212 |
| phospho-p38 MAPK(Thr180/Tyr182) Antibody | CST | #9211 |
| Beta Actin Monoclonal antibody | Proteintech | 66009-1-lg |
| Alpha Tubulin Polyclonal antibody | Proteintech | 11224-1-AP |
| HRP-conjugated Goat Anti-Rabbit IgG(H+L) | Proteintech | SA00001-2 |
| HRP-conjugated Goat Anti-Mouse IgG(H+L) | Proteintech | SA00001-1 |
| Anti-Vimentin Antibody | HUABIO | EM0401 |
| ZO-1 Polyclonal antibody | Proteintech | 21773-1-AP |
| MT-ND6 Polyclonal Antibody | Invitrogen | PA5-103954 |
| COX-1 Polyclonal antibody | Proteintech | 13393-1-AP |
| Cytochrome c Polyclonal antibody | Proteintech | 10993-1-AP |
| Anti-SOX9 Antibody | Millipore | AB5535 |
| CYP11A1 Polyclonal antibody | Proteintech | 13363-1-AP |
| Anti-DDX4 | Abcam | ab27591 |
| Goat anti-rabbit Alexa Fluor 488 | Invitrogen | A-11008 |
| Goat anti-mouse Alexa Fluor 555 | Invitrogen | A-21422 |


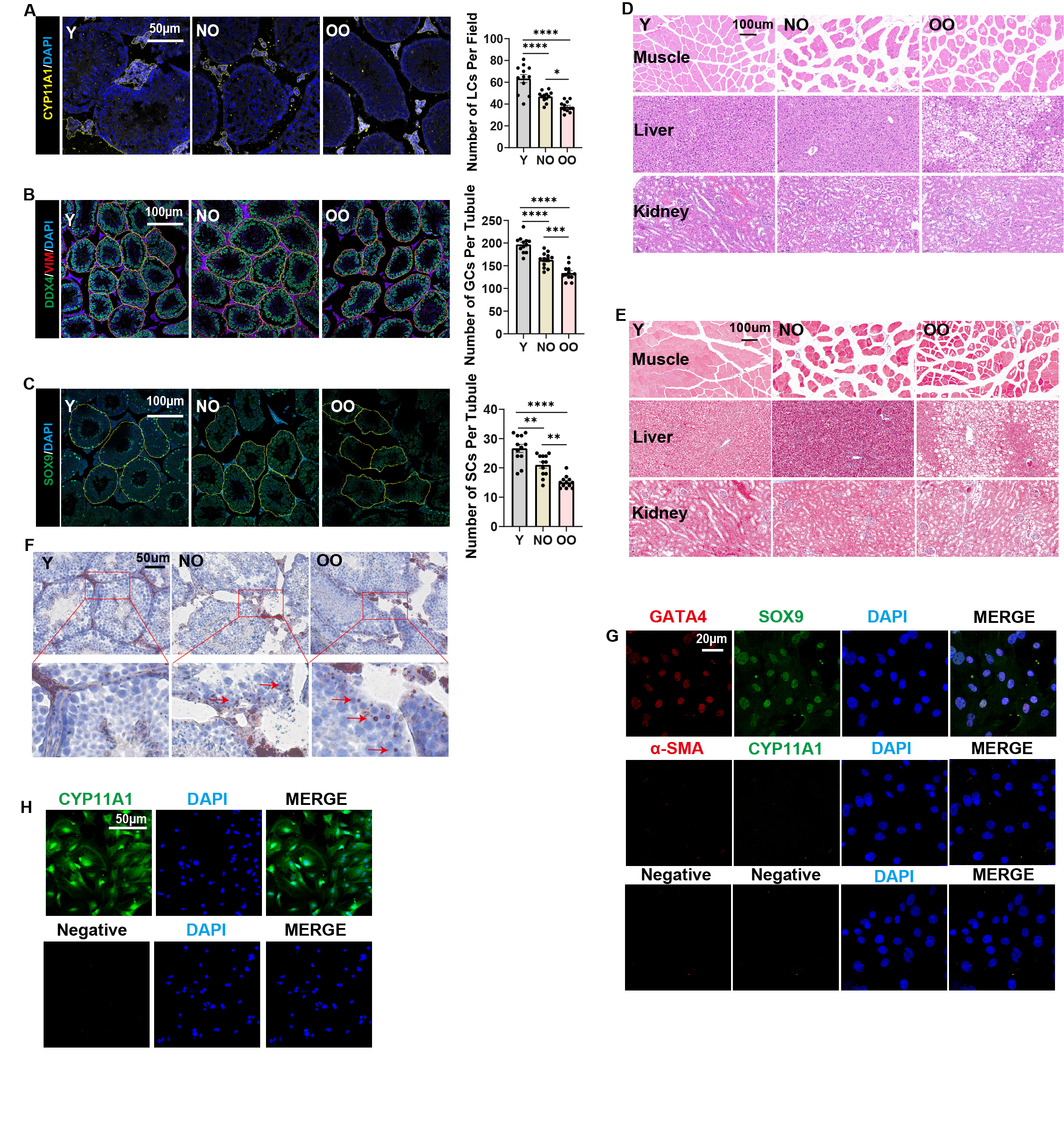


**Figure S1. Obesity Exacerbates Testicular Aging *in vivo* *and in vitro*** (A) Immunofluorescence staining for DAPI (blue) and CYP11A1 (yellow) in testicular tissue sections. n = 10. Scale bar = 50 µm. (B) Immunofluorescence staining for VIM (red) and DDX4 (green) in testicular tissue sections. n = 10. Scale bar = 100 µm. (C) Immunofluorescence staining for DAPI (blue) and SOX9 (green) in testicular tissue sections. n = 10. Scale bar = 100 µm. (D-E) Representative images of HE and Masson staining of muscle, liver, kidney tissue sections. Scale bar = 100 µm. (F) Representative images of testicular tissue sections stained with Oil Red O to visualize lipid droplets. Red arrows indicate lipid droplets. Scale bar = 50 µm. (G) Identification and purity assessment of Sertoli cells. Representative images show co-localization of markers GATA4 (red) and SOX9 (green) in Sertoli cells. Negative controls are included. Scale bar = 20 µm. (H) Representative immunofluorescence images of CYP11A1 (green) in Leydig cells stained with DAPI (blue). The negative control was processed without the primary antibody. Scale bar = 50 µm. Bars represent means ± SEM. Statistical significance was assessed using one-way ANOVA followed by Tukey’s post hoc tests or Kruskal-Wallis test with Dunn post hoc tests. *p < 0.05, **p < 0.01, ***p < 0.001, ****p < 0.0001.


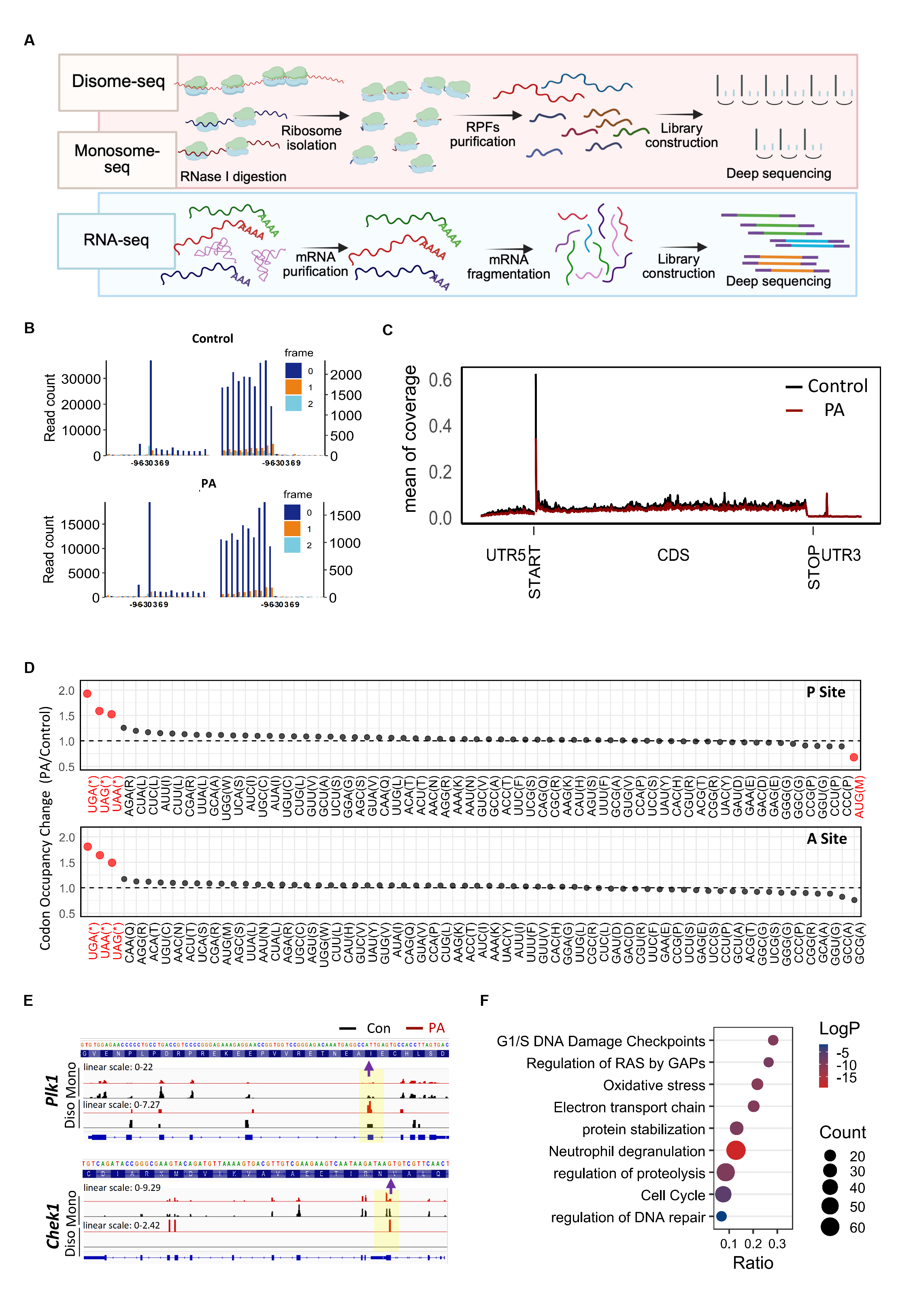


**Figure S2. ROS Induces Ribosome Stalling and Collisions** (A) Schematic diagram of the sequencing strategy. Monosome-seq (Ribo-seq) and Disome-seq were conducted simultaneously in a single experiment, with monosome (~30 nt) and disome (~60 nt) footprints being isolated separately for sequencing analysis. (B) Quality control of Monosome-seq data in control (top) and PA treated (bottom) cells. (C) Average ribosome occupancy across different mRNA regions in the PA group (red line) and the control group (black line). (D) Ribosome occupancy at individual codons in P-sites (top) and A-sites (bottom). The three stop codons (showing significant increases) and the start codon (displaying a decrease) are highlighted in red. (E) Track plot showing a significant decrease in the translation efficiency of Plk1 (top) and Chek1 (bottom) in PA-treated cells compared with control cells. Translation of Plk1 and Chek1 mRNAs was specifically arrested at regions containing D and E codons, which are indicated by red triangles. D: aspartate, E: glutamate. (F) Gene Ontology analysis of reactive pathway enrichment using the genes with high pause sites in PA treatment.


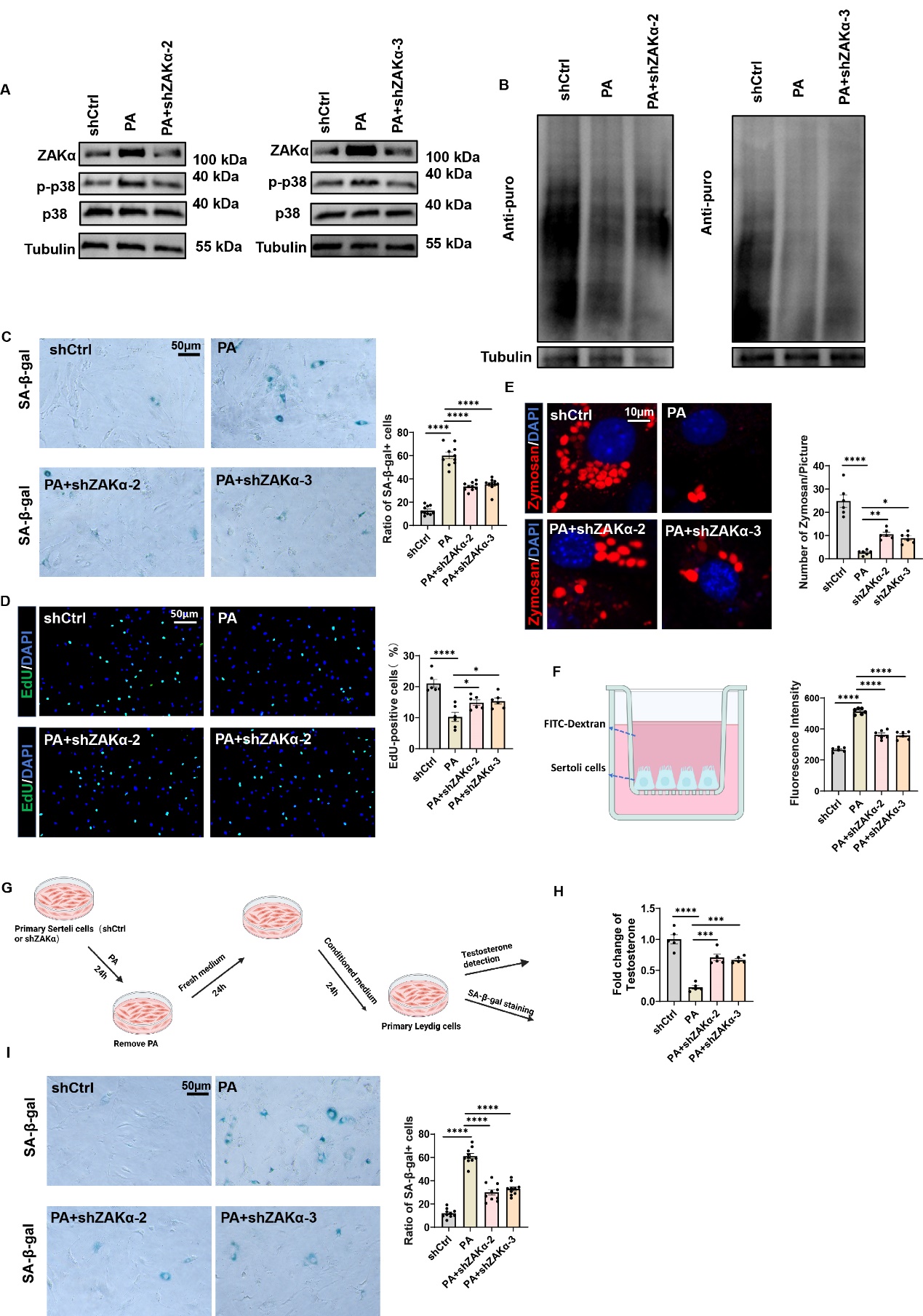


**Figure S3. ZAKα Knockdown Rescued PA-Induced Sertoli Cells Senescence** (A) Western blot shows the level of ZAKα, p38 and p-p38 in Sertoli cells from different treatment groups. (B) Western blot analysis for puromycin incorporation in Sertoli cells from different treatment groups. (C) SA-β-gal staining of Sertoli cells from different experimental groups. n = 10. Scale bar = 50 µm. (D) EdU incorporation assay in Sertoli cells from different treatment groups to measure cell proliferation. n = 6. Scale bar = 50 µm. (E) Representative fluorescence images showing Zymosan particles (red) and nuclei (blue) in different group. n = 6. Scale bar = 10 µm. (F) Schematic representation of Sertoli cells cultured with FITC-Dextran. Bar graph quantifying FITC-Dextran fluorescence intensity across experimental groups. n = 6. (G-I) Schematic workflow for evaluating testosterone secretion and SA-β-gal in Leydig cells exposed to conditioned media from treated Sertoli cells. Testosterone were quantified via ELISA. n = 5. SA-β-gal staining of Leydig cells exposed to conditioned media from treated Sertoli cells. n = 10. Scale bar = 50 µm. Bars represent means ± SEM. Statistical significance was assessed using one-way ANOVA followed by Tukey’s post hoc tests or Kruskal-Wallis test with Dunn post hoc tests. *p < 0.05, **p < 0.01, ***p < 0.001, ****p < 0.0001.


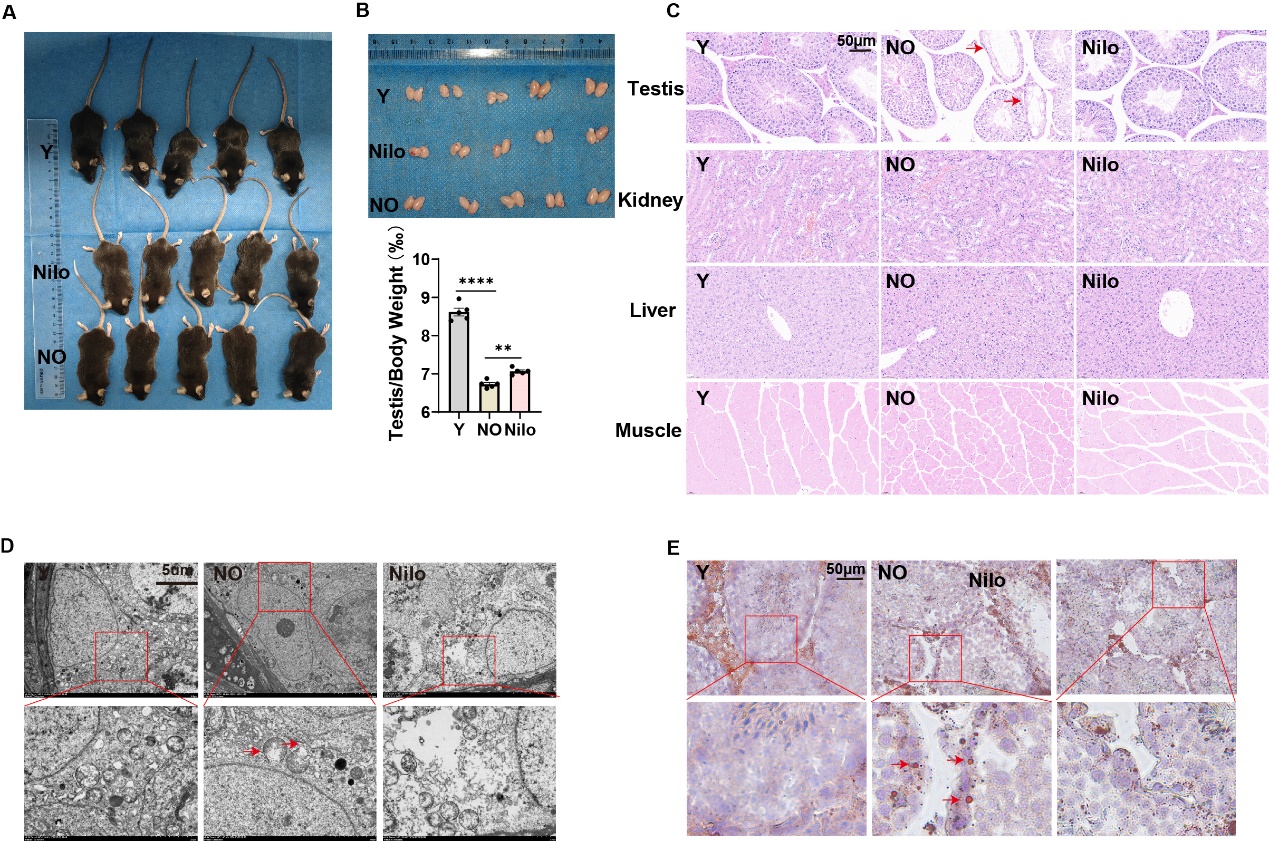


**Figure S4. The ZAKα Inhibitor Nilotinib Ameliorates the Testes of Aged Mice** (A-B) Representative images of mice and testis in the three experimental groups: Y (young), NO (naturally old), and Nilo (naturally old + Nilotinib). Testis-to-body weight ratio across experimental groups. n = 5. (C) Representative images of HE staining from testis, kidney, liver and muscle. Red arrows indicate disorganized tubules. Scale bar = 50 µm. (D) Representative images show mitochondria in Sertoli cells from mice using transmission electron microscopy (TEM). Red arrows indicate mitochondrial swelling. Scale bars = 5 µm. (E) Representative images of testicular tissue sections stained with Oil Red O to visualize lipid content. Red arrows indicate lipid droplets. Scale bar = 50 µm. Bars represent means ± SEM. Statistical significance was assessed using one-way ANOVA followed by Tukey’s post hoc tests or Kruskal-Wallis test with Dunn post hoc tests. **p* < 0.05, ***p* < 0.01, ****p* < 0.001, *****p* < 0.0001.
