# Supplementary figures and images for: Lipid Droplet‐Driven Ribosome Collisions Trigger ZAKα‐p38 Signaling to Accelerate Testicular Aging
Source: Aging Cell. 2026 Jan 2;25(1):e70359. doi: 10.1111/acel.70359 (PMC12759184; doi:10.1111/acel.70359)

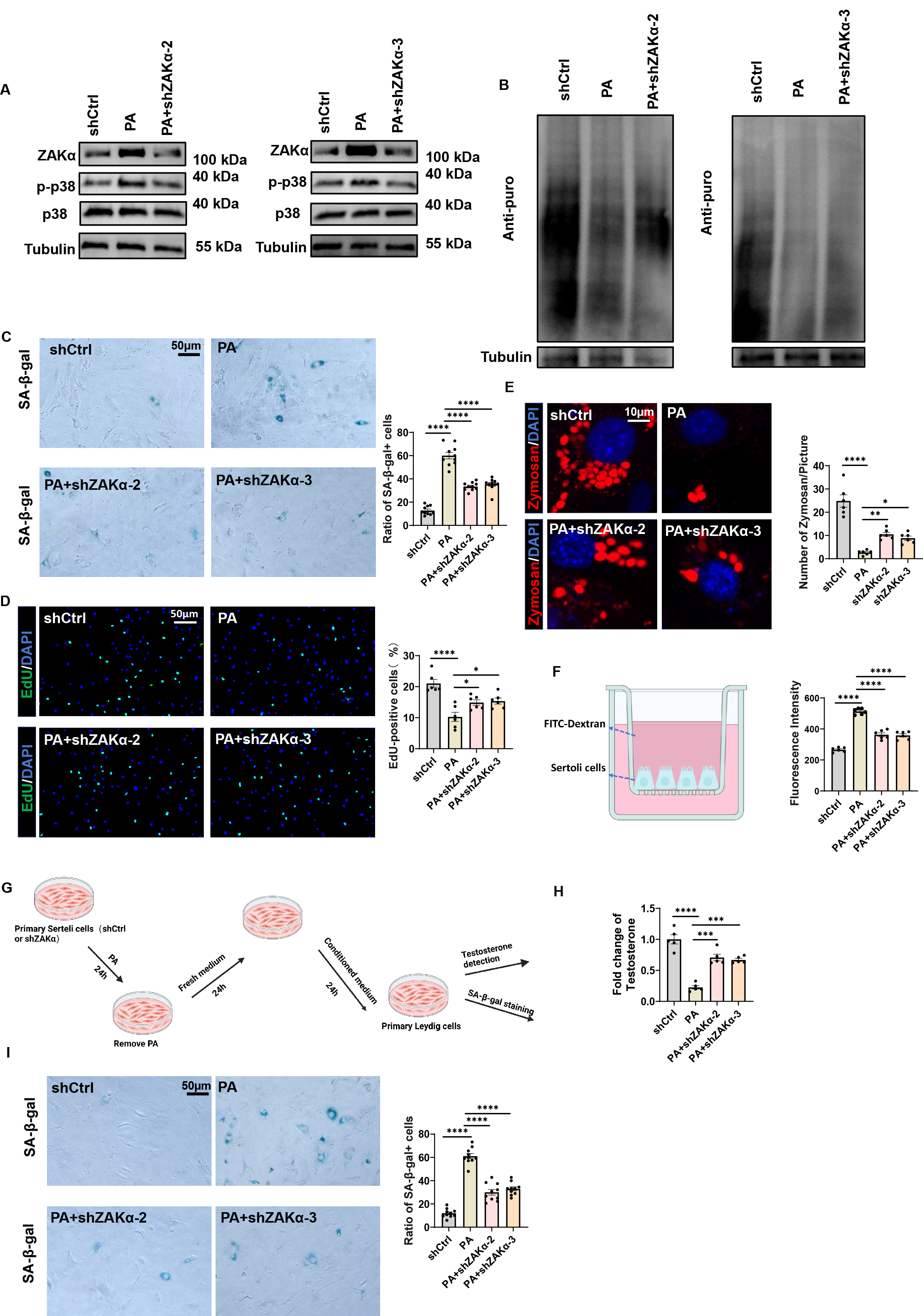

Supplement: Supplementary file 1 — Table S1: Primer sequences used for real‐time quantitative PCR analyses. Table S2: Antibody used for immunoblotting and immunofluorescence staining. Figure S1: Obesity exacerbates testicular aging in vivo and in vitro (A) Immunofluorescence staining for DAPI (blue) and CYP11A1 (yellow) in testicular tissue sections. n = 10. Scale bar = 50 μm. (B) Immunofluorescence staining for VIM (red) and DDX4 (green) in testicular tissue sections. n = 10. Scale bar = 100 μm. (C) Immunofluorescence staining for DAPI (blue) and SOX9 (green) in testicular tissue sections. n = 10. Scale bar = 100 μm. (D, E) Representative images of HE and Masson staining of muscle, liver, kidney tissue sections. Scale bar = 100 μm. (F) Representative images of testicular tissue sections stained with Oil Red O to visualize lipid droplets. Red arrows indicate lipid droplets. Scale bar = 50 μm. (G) Identification and purity assessment of Sertoli cells. Representative images show co‐localization of markers GATA4 (red) and SOX9 (green) in Sertoli cells. Negative controls are included. Scale bar = 20 μm. (H) Representative immunofluorescence images of CYP11A1 (green) in Leydig cells stained with DAPI (blue). The negative control was processed without the primary antibody. Scale bar = 50 μm. Bars represent means ± SEM. Statistical significance was assessed using one‐way ANOVA followed by Tukey's post hoc tests or Kruskal‐Wallis test with Dunn post hoc tests. *p < 0.05, **p < 0.01, ***p < 0.001, ****p < 0.0001. Figure S2: ROS induces ribosome stalling and collisions (A) Schematic diagram of the sequencing strategy. Monosome‐seq (Ribo‐seq) and Disome‐seq were conducted simultaneously in a single experiment, with monosome (~30 nt) and disome (~60 nt) footprints being isolated separately for sequencing analysis. (B) Quality control of Monosome‐seq data in control (top) and PA treated (bottom) cells. (C) Average ribosome occupancy across different mRNA regions in the PA group (red line) and the control group (b [file ACEL-25-e70359-s001.zip › ACEL_70359_f3_S3.jpg]

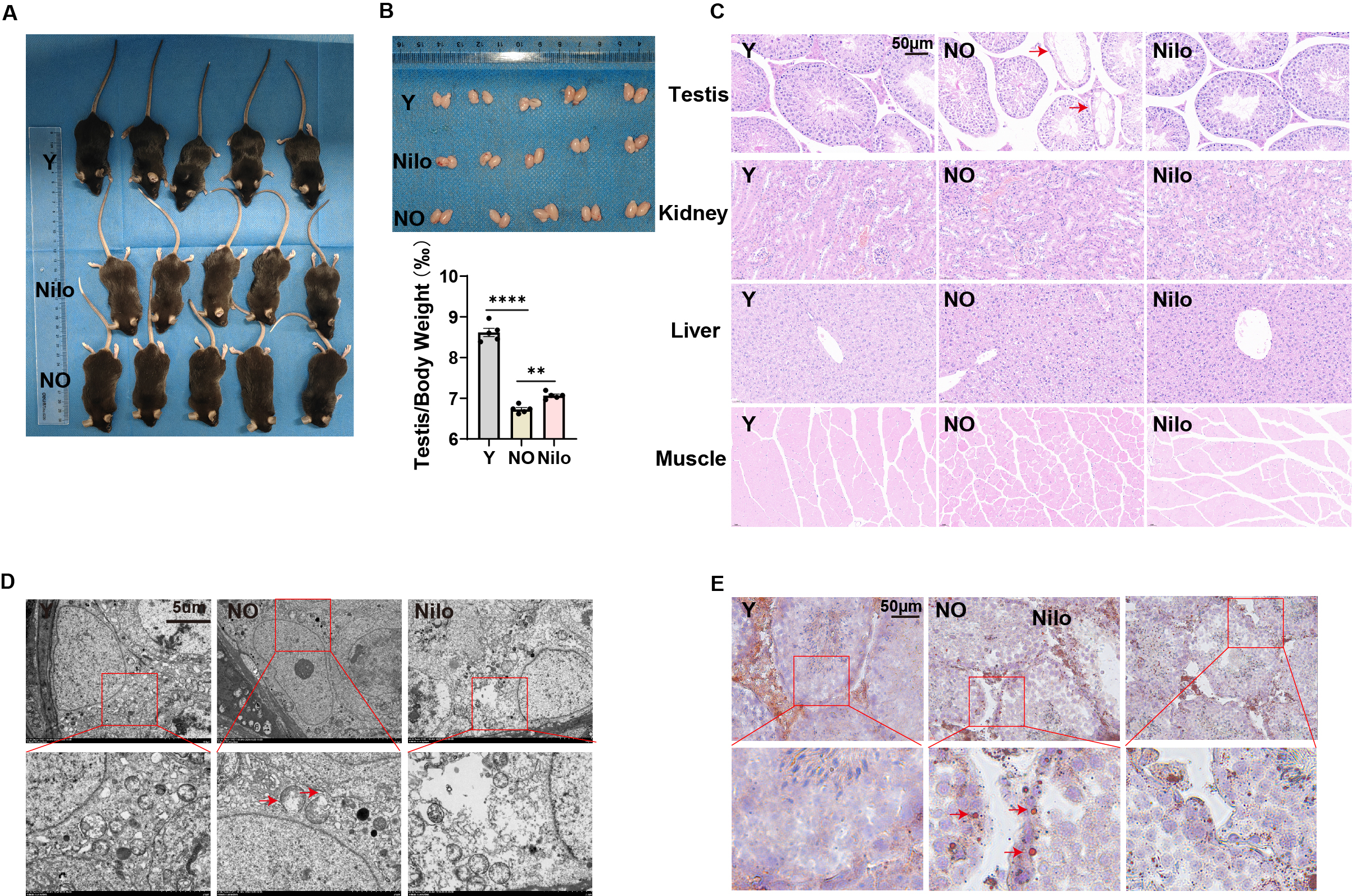

Supplement: Supplementary file 1 — Table S1: Primer sequences used for real‐time quantitative PCR analyses. Table S2: Antibody used for immunoblotting and immunofluorescence staining. Figure S1: Obesity exacerbates testicular aging in vivo and in vitro (A) Immunofluorescence staining for DAPI (blue) and CYP11A1 (yellow) in testicular tissue sections. n = 10. Scale bar = 50 μm. (B) Immunofluorescence staining for VIM (red) and DDX4 (green) in testicular tissue sections. n = 10. Scale bar = 100 μm. (C) Immunofluorescence staining for DAPI (blue) and SOX9 (green) in testicular tissue sections. n = 10. Scale bar = 100 μm. (D, E) Representative images of HE and Masson staining of muscle, liver, kidney tissue sections. Scale bar = 100 μm. (F) Representative images of testicular tissue sections stained with Oil Red O to visualize lipid droplets. Red arrows indicate lipid droplets. Scale bar = 50 μm. (G) Identification and purity assessment of Sertoli cells. Representative images show co‐localization of markers GATA4 (red) and SOX9 (green) in Sertoli cells. Negative controls are included. Scale bar = 20 μm. (H) Representative immunofluorescence images of CYP11A1 (green) in Leydig cells stained with DAPI (blue). The negative control was processed without the primary antibody. Scale bar = 50 μm. Bars represent means ± SEM. Statistical significance was assessed using one‐way ANOVA followed by Tukey's post hoc tests or Kruskal‐Wallis test with Dunn post hoc tests. *p < 0.05, **p < 0.01, ***p < 0.001, ****p < 0.0001. Figure S2: ROS induces ribosome stalling and collisions (A) Schematic diagram of the sequencing strategy. Monosome‐seq (Ribo‐seq) and Disome‐seq were conducted simultaneously in a single experiment, with monosome (~30 nt) and disome (~60 nt) footprints being isolated separately for sequencing analysis. (B) Quality control of Monosome‐seq data in control (top) and PA treated (bottom) cells. (C) Average ribosome occupancy across different mRNA regions in the PA group (red line) and the control group (b [file ACEL-25-e70359-s001.zip › ACEL_70359_f4_S4.jpg]

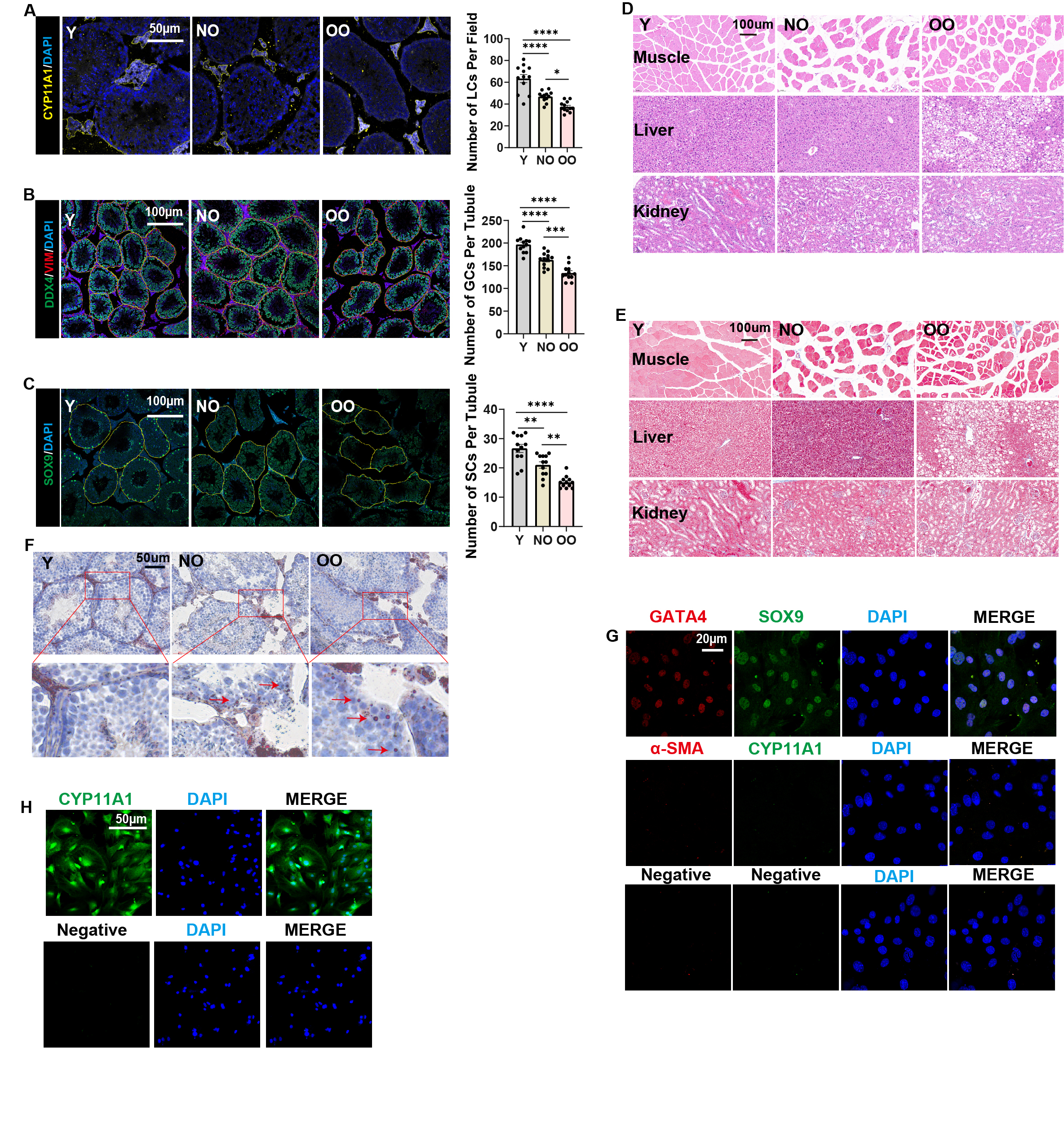

Supplement: Supplementary file 1 — Table S1: Primer sequences used for real‐time quantitative PCR analyses. Table S2: Antibody used for immunoblotting and immunofluorescence staining. Figure S1: Obesity exacerbates testicular aging in vivo and in vitro (A) Immunofluorescence staining for DAPI (blue) and CYP11A1 (yellow) in testicular tissue sections. n = 10. Scale bar = 50 μm. (B) Immunofluorescence staining for VIM (red) and DDX4 (green) in testicular tissue sections. n = 10. Scale bar = 100 μm. (C) Immunofluorescence staining for DAPI (blue) and SOX9 (green) in testicular tissue sections. n = 10. Scale bar = 100 μm. (D, E) Representative images of HE and Masson staining of muscle, liver, kidney tissue sections. Scale bar = 100 μm. (F) Representative images of testicular tissue sections stained with Oil Red O to visualize lipid droplets. Red arrows indicate lipid droplets. Scale bar = 50 μm. (G) Identification and purity assessment of Sertoli cells. Representative images show co‐localization of markers GATA4 (red) and SOX9 (green) in Sertoli cells. Negative controls are included. Scale bar = 20 μm. (H) Representative immunofluorescence images of CYP11A1 (green) in Leydig cells stained with DAPI (blue). The negative control was processed without the primary antibody. Scale bar = 50 μm. Bars represent means ± SEM. Statistical significance was assessed using one‐way ANOVA followed by Tukey's post hoc tests or Kruskal‐Wallis test with Dunn post hoc tests. *p < 0.05, **p < 0.01, ***p < 0.001, ****p < 0.0001. Figure S2: ROS induces ribosome stalling and collisions (A) Schematic diagram of the sequencing strategy. Monosome‐seq (Ribo‐seq) and Disome‐seq were conducted simultaneously in a single experiment, with monosome (~30 nt) and disome (~60 nt) footprints being isolated separately for sequencing analysis. (B) Quality control of Monosome‐seq data in control (top) and PA treated (bottom) cells. (C) Average ribosome occupancy across different mRNA regions in the PA group (red line) and the control group (b [file ACEL-25-e70359-s001.zip › ACEL_70359_f1_S1.jpg]

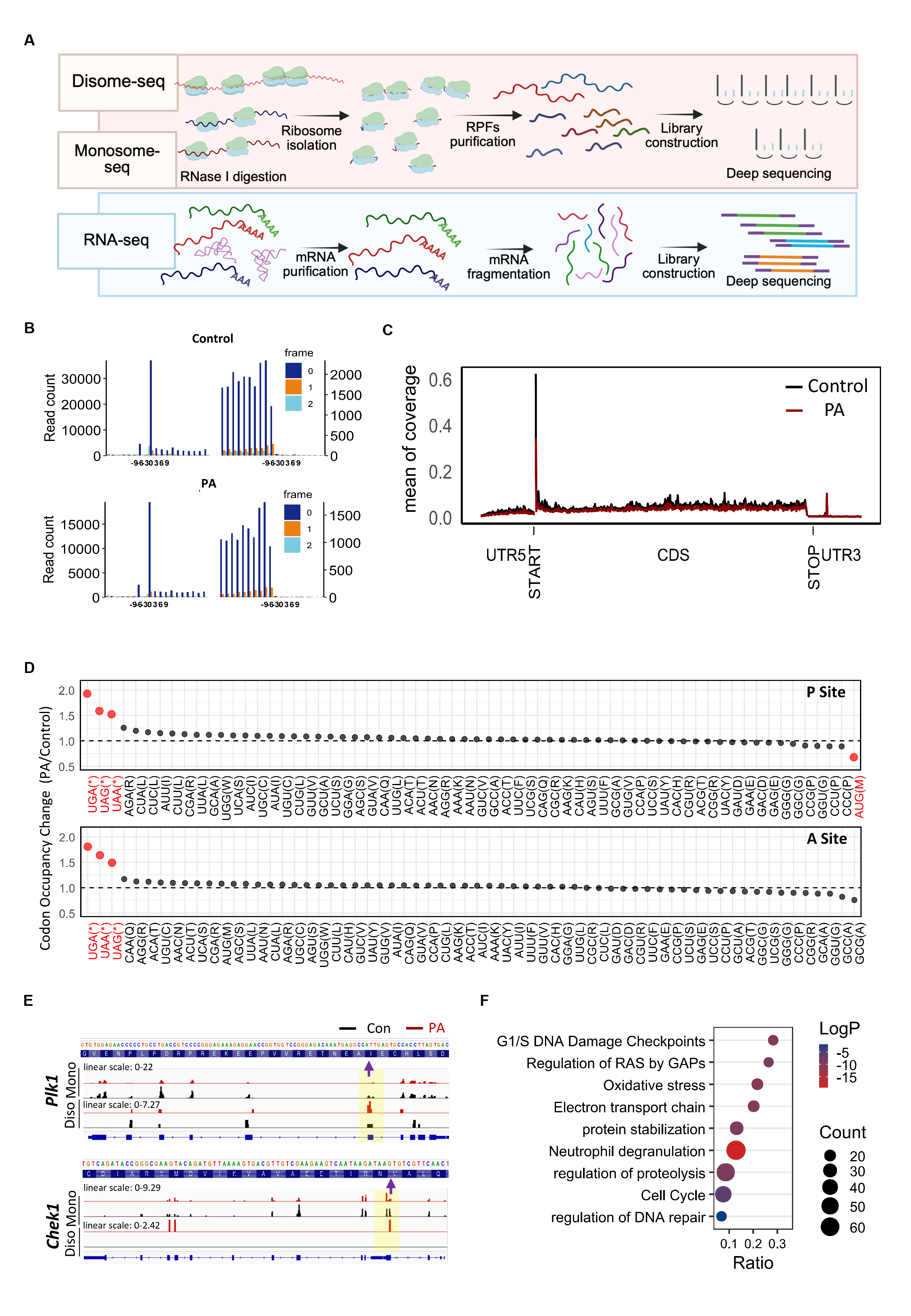

Supplement: Supplementary file 1 — Table S1: Primer sequences used for real‐time quantitative PCR analyses. Table S2: Antibody used for immunoblotting and immunofluorescence staining. Figure S1: Obesity exacerbates testicular aging in vivo and in vitro (A) Immunofluorescence staining for DAPI (blue) and CYP11A1 (yellow) in testicular tissue sections. n = 10. Scale bar = 50 μm. (B) Immunofluorescence staining for VIM (red) and DDX4 (green) in testicular tissue sections. n = 10. Scale bar = 100 μm. (C) Immunofluorescence staining for DAPI (blue) and SOX9 (green) in testicular tissue sections. n = 10. Scale bar = 100 μm. (D, E) Representative images of HE and Masson staining of muscle, liver, kidney tissue sections. Scale bar = 100 μm. (F) Representative images of testicular tissue sections stained with Oil Red O to visualize lipid droplets. Red arrows indicate lipid droplets. Scale bar = 50 μm. (G) Identification and purity assessment of Sertoli cells. Representative images show co‐localization of markers GATA4 (red) and SOX9 (green) in Sertoli cells. Negative controls are included. Scale bar = 20 μm. (H) Representative immunofluorescence images of CYP11A1 (green) in Leydig cells stained with DAPI (blue). The negative control was processed without the primary antibody. Scale bar = 50 μm. Bars represent means ± SEM. Statistical significance was assessed using one‐way ANOVA followed by Tukey's post hoc tests or Kruskal‐Wallis test with Dunn post hoc tests. *p < 0.05, **p < 0.01, ***p < 0.001, ****p < 0.0001. Figure S2: ROS induces ribosome stalling and collisions (A) Schematic diagram of the sequencing strategy. Monosome‐seq (Ribo‐seq) and Disome‐seq were conducted simultaneously in a single experiment, with monosome (~30 nt) and disome (~60 nt) footprints being isolated separately for sequencing analysis. (B) Quality control of Monosome‐seq data in control (top) and PA treated (bottom) cells. (C) Average ribosome occupancy across different mRNA regions in the PA group (red line) and the control group (b [file ACEL-25-e70359-s001.zip › ACEL_70359_f2_S2.jpg]
