# Supplementary material for: Lipid Droplet‐Driven Ribosome Collisions Trigger ZAKα‐p38 Signaling to Accelerate Testicular Aging
Source: Aging Cell. 2026 Jan 2;25(1):e70359. doi: 10.1111/acel.70359 (PMC12759184; doi:10.1111/acel.70359)
Supplement: Supplementary file 2 — Appendix S2: acel70359‐sup‐0002‐AppendixS2.docx. [file ACEL-25-e70359-s002.docx]

**Quantitative Analysis with detailed information**

**1 Senescence associated β-galactosidase (SA-β-gal) staining**

To quantify the β-gal positive area, start by converting the image to an 8-bit format. Then, proceed to Image → Adjust → Threshold, select the "Default" mode, and carefully adjust the sliders to specifically highlight the blue-stained regions in red while excluding all background tissue. Maintaining consistent threshold values across all images is essential for reliable comparative analysis. Finally, with the threshold active, configure the measurement settings to analyze the "Area" fraction and execute the measurement to obtain an accurate quantification of the stained region. Due to the varied shapes of primary cells, we use manual counting for quantifying β-gal stained positive cells.

**2 Oil Red O staining**

To quantify intracellular lipid droplets, begin by converting the image to 8-bit format. Proceed to set an appropriate threshold (Image > Adjust > Threshold) to precisely isolate the lipid droplets, ensuring only distinct circular structures are highlighted while excluding background signal. Subsequently, run Analyze > Analyze Particles with optimized parameters: set the size range to 8-Infinity pixels² to exclude tiny artifacts, and apply a circularity limit (0.60-1.00) to selectively count spherical droplets while ignoring irregular-shaped aggregates. Always verify the automated count by examining the resulting overlay against the original image, and utilize the "Display results" and "Summarize" options for comprehensive data output. For clustered droplets, we implemented the watershed separation function before particle analysis to improve counting accuracy.

**3 Lipid droplet in testicular tissue stained with Nile Red**

For quantitative analysis of lipid droplets in testicular tissue stained with Nile Red, we employ manual counting due to the characteristically large size of these organelles, which often challenges automated segmentation algorithms. The procedure is efficiently conducted using ImageJ's built-in cell counter plugin. After opening the stained image, initiate the count by selecting Plugins > Analyze > Cell Counter. Within the plugin, choose a specific marker type to label individual lipid droplets directly on the image. This manual approach allows for the precise discrimination and counting of large, distinct droplets while effectively ignoring background artifacts and aggregated clusters that could skew automated results. The final tally is automatically generated and displayed by the plugin, providing a reliable and accurate quantification method for large lipid droplets in complex tissue structures.

**4 Immunofluorescence (IF) staining of testicular tissues (ROS intensity calculation included)**

To measure immunofluorescence intensity, first convert the image to 8-bit format (Image > Type > 8-bit). Then, set an appropriate threshold (Image > Adjust > Threshold) using methods like "Default" to selectively define the specific fluorescent signals while excluding background noise. Before measurement, configure the analysis settings (Analyze > Set Measurements) by selecting "Mean gray value" and crucially enabling the "Limit to threshold" option. Finally, execute the measurement (Analyze > Measure) to obtain the average fluorescence intensity specifically within the thresholded regions, ensuring consistent threshold values are applied across all samples for reliable comparative analysis.

**5 Quantification of EdU-Positive Cells**

For automated counting of EdU-positive proliferating cells, begin by converting the fluorescent image to an 8-bit grayscale format (Image > Type > 8-bit). Apply appropriate thresholding (Image > Adjust > Threshold) using the "Default" method to selectively highlight the EdU-positive nuclei while excluding background fluorescence. Proceed to Analyze > Analyze Particles, configuring critical parameters including a size range (60-Infinity pixels²) to exclude debris and a circularity limit (0.40-1.00) to ensure accurate nuclear identification. Enable the "Display Results," "Summarize," and "Add to Manager" options to obtain the cell count and create verification overlays. For clustered nuclei, apply the watershed function before particle analysis to improve separation accuracy. Always validate automated counts by comparing the resulting overlays with original images to ensure measurement precision.
